# Conserved gene- and network-level thermal memory intervals in two divergent perennial crucifers in nature

**DOI:** 10.1371/journal.pone.0336733

**Published:** 2026-08-19

**Authors:** Yoshikazu Endo, Haruki Nishio, Oguchi Taichi, Kyoko Yamane, Victoria Faith Eseese, Clarissa Frances Frederica, Hiroshi Kudoh, Diana Mihaela Buzas

**Affiliations:** 1 Mountain Science Center of University of Tsukuba, Ikawa Forest Station 1621-2 Ikawa, Aoi, Shizuoka, Japan; 2 Data Science and AI Innovation Research Promotion Centre, Shiga University 1-1-1 Banba, Hikone, Shiga, Japan; 3 Center for Ecological Research, Kyoto University, Otsu, Shiga, Japan; 4 Tsukuba-Plant Innovation Research Center and Faculty of Life and Environmental Sciences, University of Tsukuba, Tennodai, Tsukuba, Japan; 5 Gifu University, Faculty of Applied Biological Sciences, Gifu City, Gifu, Japan; 6 Institute for Collaborative Biotechnologies, University of California Santa Barbara, Santa Barbara, California, United States of America; New South Wales Department of Primary Industries, AUSTRALIA

## Abstract

Some biological responses persist long after the initial stimulus has disappeared—a phenomenon termed cellular memory. In its long-term form, cellular memory often reflects interactions between *cis*-acting chromatin states and diffusible *trans*-acting regulators, experimentally difficult to separate *in vivo*. A key challenge is to develop a quantitative and reliable framework that captures the duration of cellular memory without prior mechanistic knowledge. The FLOWERING LOCUS C (FLC) gene illustrates this problem and opportunity: a Polycomb/Trithorax *cis*-acting chromatin switch at FLC produces bistable ON/OFF transcriptional states, while *trans*-acting factors such as VERNALIZATION INSENSITIVE 3 (VIN3) and FLOWERING LOCUS T (FT) modulate transitions between those states. While laboratory studies typically view memory as the persistence of a state after a signal disappears, annual field censuses reveal a time-integrative mode of memory where *FLC* integrates fluctuating environmental signals over past intervals. To quantify such long-term effects systematically, we formalized the thermal memory interval (TMI), the time window of past environmental cues that best predicts current gene expression—as a consistent metric. We applied TMI to the VIN3–FLC–FT module in perennial *Brassicaceae* with divergent life histories: Arabidopsis halleri subsp. gemmifera and *Eutrema japonicum*, introduced here to test generality across species. TMIs distinguished spring versus autumn FLC states and revealed distributed memory across the VIN3-FLC-FT network, with intervals from 1–150 days, extending previously reported timescales. Crucially, a regression model forecasted dynamics in an independent year, showing that integrated thermal history explains the timing of seasonal phase switching across the VIN3–FLC–FT network. While TMIs require dense time-series data and do not by themselves reveal molecular mechanism, they offer a robust, quantitative, and generalizable framework: TMIs can be extended to other genes and to alternative environmental or physiological variables, enabling direct, comparative quantification of cellular memory across genes, species, and contexts.

## Introduction

Cells have the capacity to retain information about past stimuli, a phenomenon broadly referred to as cellular memory. Such memory enables biological systems to mount appropriate responses long after the initiating signal has disappeared. In animals, immunological memory provides lasting protection against previously encountered pathogens [1]. Developmental systems also rely on cellular memory; for instance, in *Drosophila*, the specification of body segment identity during embryogenesis is stably maintained throughout the organism’s life [2]. In plants, exposure to prolonged winter cold leads to vernalization, a classic example of cellular memory that ensures flowering occurs only under favourable spring conditions [3]. Mechanistically, cellular memory, especially on long term scales, can be sustained through self-reinforcing genetic feedback loops or chromatin-based mechanisms, which often act together in complex ways that are difficult to disentangle [2].

A wide range of chromatin regulators, including Polycomb and Trithorax Group (PcG/TrxG) proteins, stabilize gene expression in bistable ON and OFF states and allow these transcriptional states to be maintained long term across cell divisions [2]. At molecular level, variation in duration of cellular memory arises from differences in how readily these states can be reversed—through both gene silencing and reactivation—which depend on the intrinsic kinetics of the chromatin regulator, the organization of gene networks at native loci and environmental/developmental contexts. As a result, cellular memory varies across loci, environmental conditions, and species [4]. While synthetic single-cell assays have capacity to measure and compare cellular memory length amongst different chromatin regulators, an experimental limitation is their observation windows, which typically span only hours to days. At the opposite extreme, developmental and physiological readouts reveal that signals can be sustained over timescales of weeks, years or even across generations [1,3,5,6] but such outcomes are indirect and system specific. Taken together, the field currently lacks a general methodological framework for quantifying the duration of long-term cellular memory to allow comparisons across loci, contexts, and organisms.

As a powerful paradigm for long-term epigenetic cellular memory, vernalization derives its strength from a multi-dimensional research framework, achieved by combining molecular genetic dissection with theoretical modelling, contrasting the distinct evolutionary versions of cellular memory in annual and perennial life histories, and translating laboratory findings within complex natural environments [7]. These diverse perspectives are yet to be fully synthesized, and they currently offer distinct observational perspectives on cellular memory.

Laboratory assays in the annual life history of *Arabidopsis thaliana* (*A. thaliana*) provide a classic model of cellular memory in vernalization, viewed as the persistence of a biological response after the initiating signal disappears. This memory is evident at the organismal level—through the maintenance of vegetative growth after embryo development and the promotion of flowering long after treatment with prolonged low temperatures mimicking winter—and at the transcriptional level, where both the active and repressed states of the central integrator of vernalization, a floral repressor *FLOWERING LOCUS C* (*FLC*) are stably maintained across cell divisions. Much like the classic PcG/TrxG gene targets involved in defining *Drosophila* segment identity [2], *FLC* exemplifies this principle of persistence: the repressed state remains once the low-temperature signal is removed [3], while the active state persists even after the initial embryonic activators are no longer present. However, while this laboratory view focuses on evidencing memory through the maintenance of states under simplified, constant temperatures, it may not fully capture the system’s behavior in natural settings where environmental signals fluctuate widely.

Beyond serving as a model for physiological memory, rare integration of experimental and mathematical approaches [8–10] in *A. thaliana* also provided one of the first mechanistic demonstrations that PcG/TrxG-modified chromatin can encode memory through bistable ON/OFF states *in cis* [8–11]. While at single-cell level *FLC* is transcriptionally ON or OFF, at the tissue level these states combine into four quadrants with distinct chromatin signatures and biological functions [7]. The “silenced” and “active” [3,12] FLC expression states are maintenance states and correspond to all cells being transcriptionally OFF or ON, referred to here as the FLC Minimum and FLC Maximum states, respectively. Between these poles lie the “dialling” phases—intermediate populations where reversible switching between states enables quantitative shifts in total transcript levels. During dial-down, prolonged cold progressively biases switching toward the OFF state through the sequential action of *trans*-acting regulators: *VERNALIZATION INSENSITIVE 3* (*VIN3*; [13]) induction during sustained cold enables Polycomb-mediated silencing and the digital accumulation of OFF states, while *FLOWERING LOCUS T* (*FT*; [14]) and associated flowering-time pathways contribute to later-stage reinforcement and consolidation of repression. Conversely, during dial-up, switching is biased toward ON states, a process evidenced in two distinct contexts. In the rare ecotype, *FLC* reactivates during vegetative growth through cell-autonomous OFF-to-ON transitions, revealing the intrinsic bistability of the *cis*-chromatin [15]. Similarly, genetic and chromatin analyses indicate that the same process in embryos—traditionally described as “resetting” [16,17]—actually reflects staged *de novo* activation rather than a simple reversal of silencing: early embryonic activation initiates the resolution of OFF states, which are subsequently amplified and stabilized by developmental inputs [18–20]. Consequently, the chromatin switch at *FLC* expands the classical view of memory as simple persistence; instead, it reveals a system that actively tracks state changes across the life cycle, where dial-up and dial-down are inseparable phases of the same switch modulated by temporally distinct *trans*-acting regulators [7]. This ‘Season-Meter’ framework unifies research that has largely evolved along dispersed observational axes: laboratory studies of ‘dial-down’ repression [3,13], embryonic studies of ‘resetting’ [16–19] and ecological studies of perennial cycles [7,21–23].

A series of studies extended vernalization research from controlled laboratory assays to natural field conditions and from annual to perennial life histories [21–24]. Among these, Aikawa et al. (2010) identified a distinct form of *FLC*-based cellular memory, defined not by persistence after signal disappearance but by the integration of fluctuating temperature signals over time [7,22]. Aikawa et al. (2010) provided three key insights. First, the evergreen perennial *Arabidopsis halleri subsp. gemmifera* (*A. halleri*, hereafter, and *Ahg* as a prefix of gene name) allows continuous monitoring of *FLC* gene expression throughout the annual cycle in dividing leaf cells, revealing a cyclic reiteration of *FLC* maintenance and dialling states across seasons. Second, despite multiple environmental cues influencing flowering in perennials [25], temperature alone explained ~80% of *AhgFLC* expression variance when averaged over the preceding six-week window, demonstrating that *FLC* integrates temperature across time. The strong predictive power of a single factor highlights an exceptional form of cellular memory: integration of a fluctuating environmental signal over a defined previous temporal window. Third, Aikawa et al. (2010) introduced an empirical approach to capture this property by regressing gene expression against past temperature windows.

Here, we formalize the Thermal Memory Interval (TMI) as a transferable metric which quantifies the timescale over which past temperature history predicts current transcriptional states. Rather than seeking to dissect molecular mechanisms or define functional roles of individual loci, this study leverages the experimental advantages of natural populations of evergreen perennials growing in their native environments to evaluate whether TMI represents a quantifiable property at both gene and network scales. Specifically, we asked whether TMIs can be measured robustly and whether they provide biologically informative insights into chromatin stability, transcriptional responsiveness, and delayed gene expression over seasonal timescales. To establish this foundation, we introduced *Eutrema japonicum* (*E. japonicum*), a divergent evergreen perennial relative of *A. halleri*. The *E. japonicum* genome was recently sequenced [26,27] and revealed an allotetraploid structure where core flowering regulators—including *EjFLC*, *EjVIN3*, and *EjFT*—are present as a single locus per subgenome. This species has a strict requirement for vernalization to initiate flowering [28,29]: direct evidence for this requirement is seen in laboratory settings, where specific cold treatments efficiently induce flowering [29], and in agricultural observations where plants remain entirely vegetative throughout the following year if they fail to perceive a sufficient seasonal cooling window [28]**.** Comparative analysis is informative because unlike the atypical life history of *A.halleri*, where all meristems transition to reproductive fate before reverting to vegetative growth, *E. japonicum* exemplifies a more typical perennial strategy, with only some meristems becoming reproductive during winter while others remain vegetative to sustain growth into the next cycle. We first confirmed that the *FLC* orthologues in *E. japonicum* represses flowering and that prolonged cold exposure relieves this repression. To evaluate TMI framework, we pursued three complementary approaches. First, we combined high-resolution gene expression data with environmental temperature records to calculate TMIs and assess their predictive power. By developing a cross-year predictive model to forecast dynamics in an independent year, we tested the robustness of the TMI as a reproducible biological property and evaluated whether the integrated history of past temperatures was sufficient to explain the timing of seasonal gene expression phase switching observed across the network in nature. Second, we compared TMIs across the two divergent perennials, applying them as general ecological parameters to classify cellular memory types and identify conserved features. Analysis of these data, together with chromatin and transcriptional profiling of *EjFLC* in autumn and spring, demonstrated that TMIs reliably differentiate *FLC* dialling states and provide predictive insight beyond direct molecular measurements. Finally, transfer experiments tracking the EjVIN3–EjFLC–EjFT network dynamics under non-native but natural environments revealed robust network configuration, while also showing that temperature variation experienced up to six months prior left a detectable mark. Notably, the thermal memory interval of *FT* exceeds that of *FLC*, a feature conserved across divergent perennial life histories, extending the known range of thermal memory from six to 21 weeks and suggesting cumulative information flow within the VIN3–FLC–FT network. Together, these approaches position TMIs as a tractable ecological parameter for quantifying time-integrative memory across genes, networks, and species.

## Materials and methods

**Study sites and phenotyping.** We selected a natural population of *E. japonicum* situated in the Ikawa Forest Station of the Mountain Science Center,University of Tsukuba, in Shizuoka, Japan at 35°13’ N, 138°14’ E, 710 m in altitude, where plants grow under partial canopy cover in a forest understory environment, in a flat area with water running down during restricted times of the year. For *A.halleri*, the natural population location has been described [22,23,30]. For the September and October Tsukuba transfer experiments, *E. japonicum* plants were placed outside in Tsukuba, Ibaraki at 36°05′ N, 140°14′ E, 26 m altitude. *E. japonicum* plants scored as “flowering” included visible flower buds and all stages of bolting. A minimum of 12 random plants with similar root diameter (about 1–1.5 cm) was included in each sampl

**Plant and tissue sampling.** For *E. japonicum*, a repository of all samples is included in S1 Table. About 200–300 mg of leaf tissue was pooled from at least six different plants for each of the three biological replicates and immediately placed on dry ice (Ikawa series 2016–2018) or at −80**°**C (Tsukuba series 2018–2019), except for the 2021–2022 series, where plants (subterranean stem portion and all aerial parts) were first placed at 4**°**C for a maximum of 24 h during transport to the laboratory, before freezing at −80**°**C. For the 2021–2022 series, *E. japonicum* stems of about 1- 1.5cm in diameter were detached in Ikawa from 12 plants per treatment, transported to the laboratory, and planted into cactus soil mix pots (S8 Fig); the first sampling was performed when new growth was confirmed approximately four weeks after transfer. Sampling was done during 12.00–13.00 (Tsukuba) and 14.00–16.00 (Ikawa). For temperature transfers, two sets of re-potted plants for each “winter” (December 19th, 2018) and “spring” (March 19th, 2019) were placed into growth chambers at 4**°**C or 23**°**C and further divided into 14- and 28-days subsets. Sampling in *A.halleri* was performed as previously described [22,23].

**Transgenic *A. thaliana* assays.** cDNA prepared from *E. japonicum* RNA sampled on September 21, 2016, was amplified with attb1 and attb2 oligos using PrimeSTAR HD DNA polymerase (Takara, R010A). The products were recombined into pDONR221 (Invitrogen, 12536017) using the Gateway BP Clonase II enzyme mix (Invitrogen, 11789−20) and 12 clones were sequenced. One *EjFLC-A* and *EjFLC-B* clone was recombined into the pB2GW7 binary vector [31]. The final plasmids were transformed into *Agrobacterium tumefaciens* strain *GV3101* and transformed into the *flc3* mutant in the Col-0 background using floral dip [32]. Flowering was scored as days to flower or DNF (Did Not Flower), when no flower emerged within the first 100 days from planting, in 50 control and transgenic T0 transformants for each construct.

**Genomic and cDNA sequencing, RNA extraction, quantitative RT-PCR and Chromatin Immunoprecipitation.** To determine gene copy numbers, BLAST searches were performed using *Arabidopsis thaliana* sequences from TAIR (AT5G10140.1 for *AtFLC*, AT5G57380 for *AtVIN3*, and AT1G65480 for *AtFT*) as queries against the haplotype-resolved assembly of the *E. japonicum* ‘Magic’ genome [26]. Results confirmed that *EjFLC* and *EjVIN3* are each present as a single locus per subgenome, located on chromosomes A05 (Accession: CP163465.1) and B05 (Accession: CP163472.1). Similarly, *EjFT* exists as a single locus per subgenome on chromosomes A06 (Accession: CP163466.1) and B06 (Accession: CP163473.1) [26,27]. For *E. japonicum*, RNA was extracted using RNeasy Plant Mini Kit (Qiagen, Hilden, Germany), and cDNA was prepared from 2 µg of RNA using Superscript III (Invitrogen 18080051). cDNA clones from six different months of the year were sequenced at Eurofins Genomics. Q-RT PCR and ChIP q-PCR were performed on three biological replicates, each with three PCR repeats, and carried out using Toyobo THUNDERBIRD SYBR qPCR Mix (QPS-201) and ABI7900. Expression levels were calculated using the ΔΔCT method [33], and for the ChIP experiment, the absolute DNA amount was quantified using input DNA for the standard curve. ChIP amplicons were designed for regions with 100% sequence identity between *EjFLC-A* and *EjFLC-B*. For *A.halleri*, all procedures were performed as previously described [22]. All oligonucleotide sequences are listed in S2 Table.

**Statistical modelling.** Temperature records were obtained from the nearest Japan Meteorological Agency station (35°13′ N, 138°13.3′ E 755m altitude in Ikawa, Shizuoka; 36°6.2′ N, 140°13.2′ E, 26m in Tsukuba, Ibaraki for *E. japonicum* and 34°59.9′ N, 134°59.8′ E, altitude 72m, Nishiwaki, Hyogo Prefecture, Japan for *A. halleri*). Simple moving averages (SMA) of temperatures were calculated for the time windows of the previous 1 d − 150 d from each sampling date. Gene expression was modelled using the SMA of temperature for each time window by linear regression using the lm function in R v4.1.1. The R^2^ value was calculated for the SMA of the temperature for each time window. One thousand bootstrap data sets of gene expression and SMA of temperature were created to estimate the median and 95% intervals of the R^2^ values. For all bootstrap datasets, we calculated the median and 95% intervals of the best SMA period, which explained the gene expression at the highest R^2^ value.

The intervals used to calculate the SMA were from two seasons, as follows: for *E. japonicum*, September 7, 2016 to February 22, 2017, for *EjFLC* dial-down and March 8, 2017 to May 31, 2017, and from March 14, 2018 to May 23, 2018, for *EjFLC* dial-up; for *A.halleri* November 6, 2012 to February 19, 2013, and November 5, 2013 to February 18, 2014, for *AhgFLC* dial-down and March 7, 2013 to May 28, 2013, and from March 4, 2014, to May 27, 2014, for *AhgFLC* dial-up.

## Results and discussion

### The divergent perennial life histories of *Arabidopsis halleri* subsp. *gemmifera* and *Eutrema japonicum* provide distinct experimental opportunities

**Fig 1:**
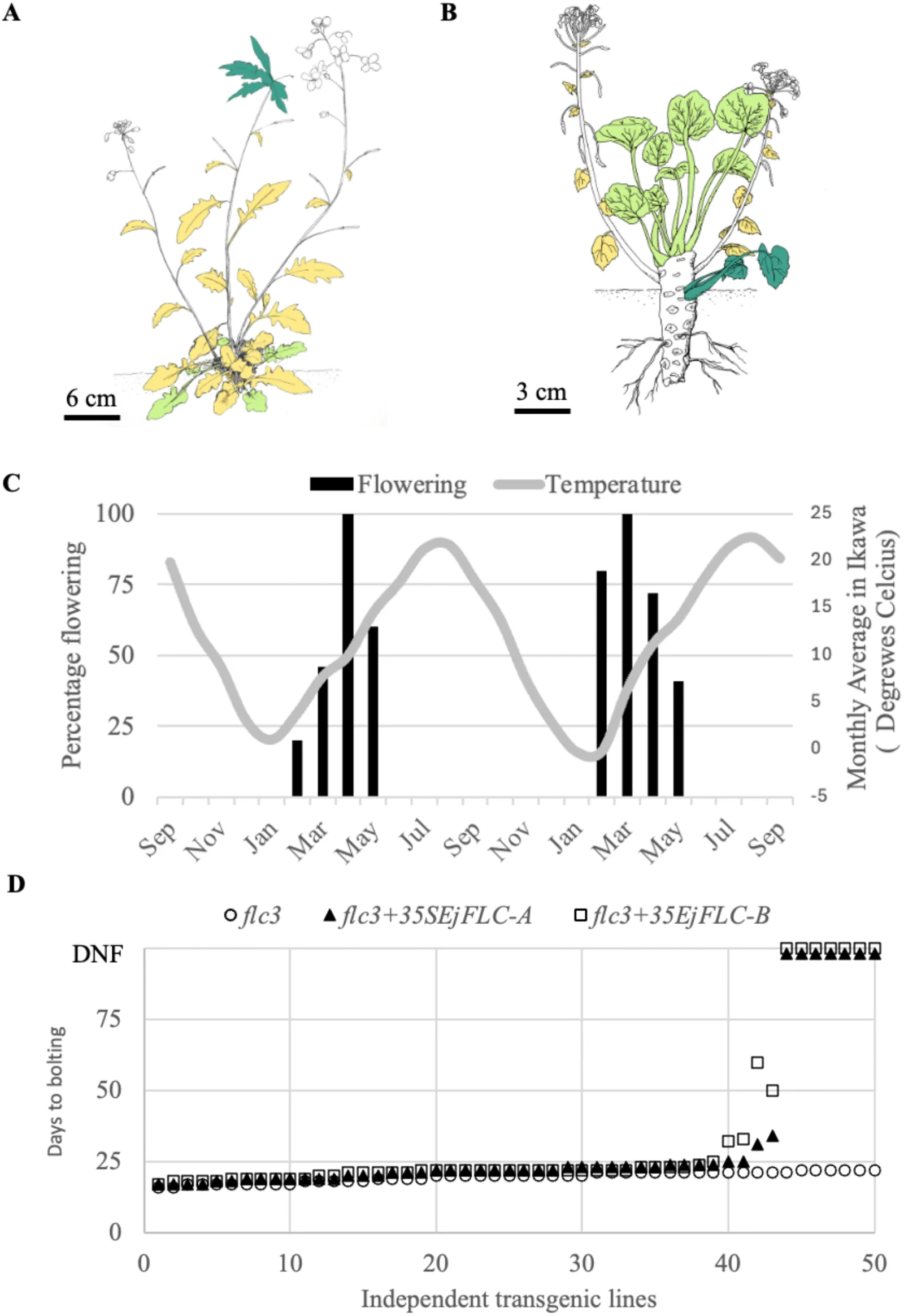
Perennial life history in two divergent *Brassicaceae* perennial life histories, *Eutrema aponicum* vernalization response and transgenic assay in *Arabidopsis. thaliana.* (A) General *A.rabidopsis halleri* subsp. *gemmifera* biology. Rosette leaves (pale green) largely disappear during the peak spring flowering season as vegetative growth converts into reproductive growth. At this time, most of leaves at ground level are basal cauline leaves (yellow). Later, all reproductive meristems revert to vegetative growth (determinate type), giving rise to aerial rosettes (dark green) for clonal propagation. This illustration is a composite image where structures from different developmental stages are drawn together for conceptual clarity; this configuration is not found in natural settings at any one time. (B) General *E. japonicum* biology. Rosette leaves (pale green) emerge from the central uppermost tiers of the stem. Cauline leaves (yellow) on inflorescence stems emerge from distinct lower tiers and are thus easily distinguished spatially from rosette leaves. Another tier level at the soil interface maintains some meristems in vegetative phase (indeterminate type) to produce new rosette leave (dark green) for clonal propagation. Photographs of flowering *A. halleri* and *E. japonicum* can be found in S1 Fig (C) *E. japonicum* flowering interval plotted against the monthly average temperature in Ikawa. (D) Complementation assay with two *E. japonicum* *FLC* genes (*EjFLC-A* and *EjFL-B*) in the *A* *thalian*a *flc3*Col-0, estimated by flowering time in 50 independent transgenics, plotted in ascending order of days to flower, ending with plants which Did Not Flower (“DNF) during the 100 days of the experiment.

Tracking long-term cellular memory depends on the ability to follow gene expression ideally in the same cell lineage and over natural timescales. Here, we focus on two perennial *Brassicaceae* with divergent life histories, *A.halleri* and *E. japonicum* (Fig 1A, 1B), each offering distinct experimental opportunities. *AhgFLC* is already well characterized in natural populations [21–24,34]; however, meristems in *A.halleri* are atypically indeterminate: all vegetative meristems transition to reproductive growth after winter and then revert to vegetative fate in spring, producing aerial rosettes for the next cycle (Fig 1A, S1 Fig). This unusual meristem biology is expected to be tightly linked with the kinetics of *AhgFLC* dial-up [21,34] and, by extension, with the dynamics of all four FLC regulatory phases [7]. To broaden this framework, we introduced *E. japonicum*, which is also an evergreen perennial *Brassicaceae* relative but has so far been little explored in vernalization studies. *E. japonicum* represents a more typical perennial system in that it has determinate meristems: vernalization triggers flowering in only a subset of meristems, while others remain vegetative to sustain growth into the next cycle. Moreover, in *E. japonicum* the meristems giving rise to reproductive growth are located in the upper tiers and are therefore spatially distinct from vegetative meristems (Fig 1B). This clear distinction between rosette and cauline leaves, present in *E. japonicum*, disappears in *A.halleri* towards the end of spring. *A.halleri* produces primarily cauline leaves in April and May, which limits rosette leaf availability for sampling during this period [22,23]. Therefore, *E. japonicum* permits continuous, year-round sampling of one lineage, i.e., dividing rosette leaves—a feature not possible in *A.halleri*—which provides a more direct route to monitoring the four regulatory phases of FLC across a full annual cycle. By comparing the two species, it becomes possible to distinguish more general properties of long-term cellular memory from aspects of *FLC* regulation that depend on meristem biology.

### Flowering in a *E. japonicum* natural population is synchronized to early spring and it is inhibited by *FLC* orthologues

To investigate whether *E. japonicum* exhibits a vernalization response under natural conditions, we first determined the flowering interval in a selected natural population of *E. japonicum* from Ikawa, Shizuoka. In agreement with previous studies [35], differentiated flower buds became visible in February, the peak of flowering was in March and flowering ended by early May; additionally, plants remained vegetative outside this interval of the year (Fig 1C). Therefore, floral buds were initiated under the short days immediately following the coolest time of the year, in two consecutive years (Fig 1C). The finding that prolonged low temperatures, but not long days, are critical for flower initiation in *E. japonicum* is consistent with other perennial crucifers [36,37], including *A.halleri* [21,22]. Therefore, these observations strongly suggest that *E. japonicum* undergoes vernalization in its natural environment to synchronize flowering to late winter/early spring.

To characterize the *FLC* orthologues in *E. japonicum* in the Ikawa ecotype, we first sequenced cDNA clones spanning the start and stop codons of *EjFLC* sampled throughout the year. Our sequence analysis identified two types of clones with six single nucleotide polymorphisms resulting in two amino acid differences. The BLAST searches against the “Mazuma” ecotype [26] indicated that they correspond to the A and B subgenomes (S2A Fig). To test if *EjFLC* can inhibit flowering in the reference *A. thaliana*, complementation tests were performed with *EjFLC-A* and *EjFLC-B* in loss-of-function *A. thaliana* *flc3*Col-0 mutant. Flowering was delayed to a different extend in most primary transgenics of both flc3Col-0 35S*:EjFLC-A* and *flc3*Col-0 35S:*EjFLC-B*. While in the majority of independent transgenic lines the T-DNA insertions may have generated insufficiently high expression and the flowering delay was not present or weak (Fig 1D, lines 13–39), other lines clearly delayed flowering and, furthermore, 14% of *flc3*Col-0 35S:*EjFLC-A* and 18% of *flc3*Col-0 35S:*EjFLC-B* did not flower in the first 100 days of the experiment (Fig 1D, lines 40–50). This data indicated that both *EjFLC-A* and *EjFLC-B* are similarly potent floral inhibitors, as expected from high amino acid identity between *AtFLC* and *EjFLC* (S2B Fig). The high sequence similarity between *EjFLC-A* and *EjFLC-B* prevented us from quantifying individual *EjFLC* mRNAs. To understand if *EjFLC* genes are differently regulated throughout the year, we determined how *EjFLC-A* and *EjFLC-B* are represented in cDNAs prepared from samples from six months **(**S3 Fig**)**. As we did not detect any major bias of one gene or the other we performed quantitative analysis of the sum, labelled “*EjFLC”* from here onwards.

### Two divergent *Brassicaceae* perennial life histories mirror multiple gene expression characteristics

The four-quadrant *FLC* expression cycle in *A.halleri* reflects a temperature memory system year-round [21]. To test whether this pattern represents a broader feature of *Brassicaceae* perennials, we conducted a high-resolution, two-year expression analysis of the three core vernalization pathway orthologues genes, *VIN3*, *FLC*, *FT* in the natural populations of *E. japonicum* from Ikawa, Shizuoka against the previously studied *A.halleri* natural population from Hyogo [22–24] (Fig 1C).

**Fig 2:**
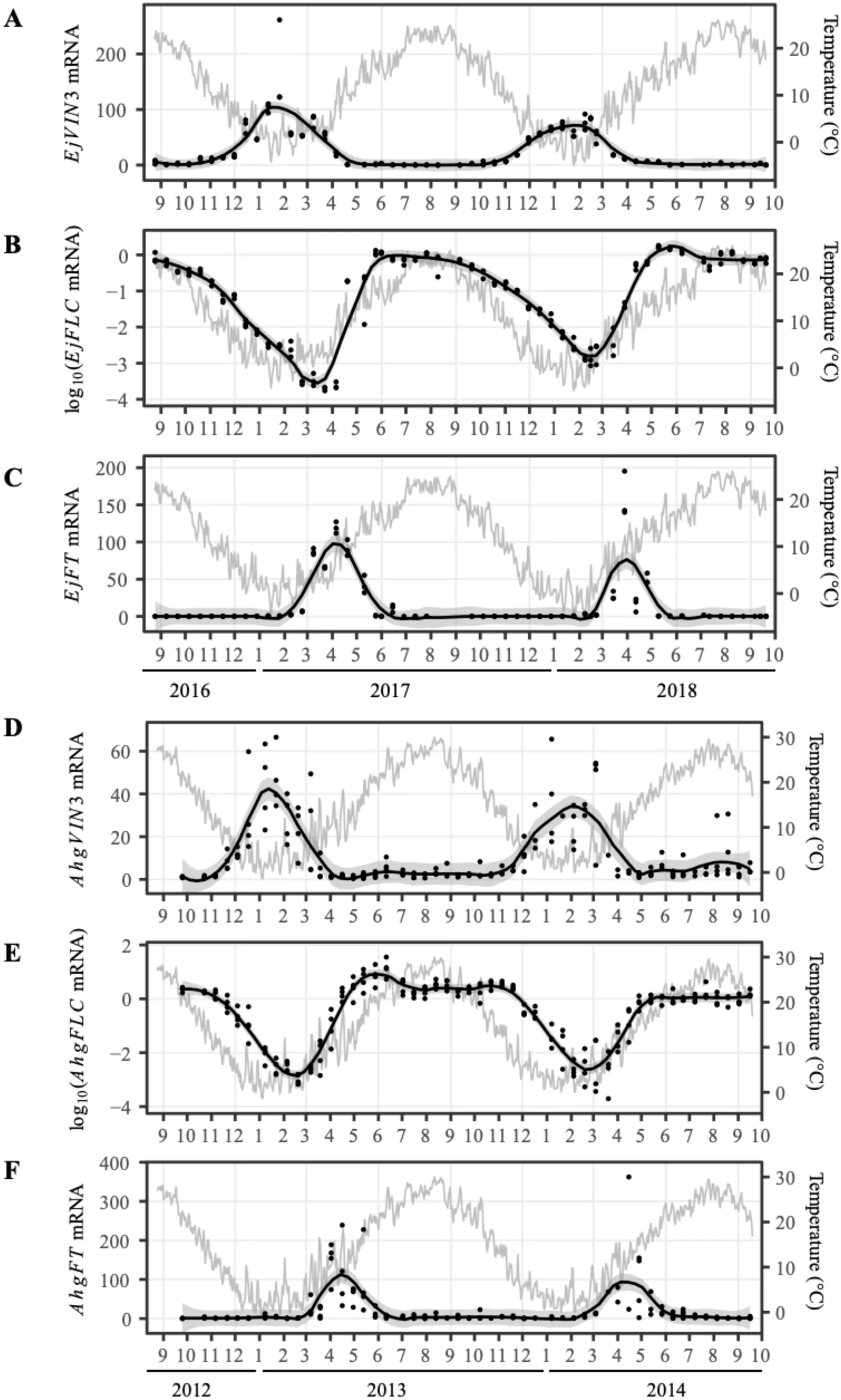
Two-year biweekly dynamics of *VIN3*, *FLC*, and *FT* mRNA levels in natural populations of two divergent perennial species. (A–C) Relative mRNA levels of *EjVIN3* (A), *EjFLC* (B), and *EjFT* (C) in *Eutrema japonicum*, quantified using the 𝜟𝜟CT method. (D–F) Relative mRNA levels of *AhgVIN3* (D), *AhgFLC* (E), and *AhgFT* (F) in *Arabidopsis. halleri* *subsp* *gemmifera*, quantified using the 𝜟𝜟CT method. In (A–F), dots, solid lines, and shaded regions represent observed mRNA levels, LOESS-fitted values, and 95% confidence intervals of the LOESS regression, respectively. Gray lines represent daily mean air temperature. Biological replicates per time point were 3 for *E. japonicum* and 3.58 ± 0.77 (mean ± SD) for *A. halleri*. *EjPP2A3* (A–C) and *AhgACT2* (D–F) were used as internal controls.

Despite having divergent life histories, both species displayed striking conservation of multiple expression features. First, both *AhgFLC* and *EjFLC* expression levels spanned up to over 10^3^-fold under natural field conditions. By spring, steady-state levels dropped by approximately 10^3^-fold (Fig 2B and 2E). This large dynamic range in the field far exceeds the ~ 10-fold change typically observed after saturating vernalization intervals in laboratory experiments, regardless of sample type or life history [12,38]. Indeed, in *A.thaliana* the reduction in *AtFLC* levels under field conditions in a winter annual ecotype [39] was also of a greater magnitude than under laboratory conditions, indicating that a large amplitude of *FLC* expression under natural conditions is conserved across annual and perennial life histories. These results suggest considerable differences in transcription rate and or mRNA degradation rate may occur between laboratory and field conditions. Alternatively, consistent with ON/OFF transcriptional states, it could be that *FLC* becomes activated in a larger fraction of dividing cells of leaves under natural conditions. This elevated transcription level would facilitate the observed graded response over the long duration in which plants encounter low temperatures in autumn and winter (Fig 2B, September to March; 2E, November to March).

Second, the spline curves of *AhgFLC* and *EjFLC* gene expression are tilted, revealing two asymmetries: between the maintenance quadrants (*FLC* maximum and *FLC* minimum) and between the dialling quadrants (*FLC* dial-up and *FLC* dial-down) (Fig 2B, 2E). We observed that *FLC* expression remained at a minimum for approximately 5 weeks in *E. japonicum* and 6 weeks in *A.halleri*. These durations are considerably shorter than the maximum *FLC* expression phases, which lasted about 14 weeks in *E. japonicum* and 22 weeks in *A.halleri*. These timelines also contrast sharply with controlled vernalization assays, which typically show an FLC minimum lasting for example about 7 days in the *Arabis alpina* perennial [40] and in *A.halleri* [41]. These results suggest that, counterintuitively, fluctuating environmental conditions in nature may promote longer term stability of *FLC* maintenance states compared to the constant conditions of laboratory settings.

Surprisingly, *FLC* dialling states are asymmetrical between autumn and spring, even though seasonal temperature ranges are comparable (Fig 2B, 2E). In *E. japonicum*, *EjFLC* dial-down lasted 18 weeks, whereas dial-up took only 9 weeks. A similar bias was observed in *A.halleri* (12 vs. 6 weeks). Previous vernalization assays in both annual and perennial plants suggest a similar tendency to asymmetry in *AtFLC*, *AaFLC*, *AhgFLC* expression [16,22,38,42] but such comparisons are confounded by measurements across different cell lineages. For example, the *AtFLC* dial-down takes place in leaves while *AtFLC* dial-up in embryos [19]. Our *E. japonicum* analysis under natural conditions overcomes this limitation by tracking both processes in the same cell lineage, namely rosette leaves. These results provide the first direct evidence that *FLC* dial-up and dial-down are not simply equivalent mirror-image all-or-none activation and silencing events but instead display a seasonal bias. This supports the view that *trans*-acting inputs modulate the *cis*-encoded *FLC* chromatin switch throughout the year [7].

Third, annual expression patterns of *VIN3*, *FLC*, and *FT* formed a consistent regulatory sequence across species. In both *A.halleri* and *E. japonicum*, *VIN3* peaked before *FLC* Minimum, and *FT* peaked immediately afterward (Fig 2). This VIN3–FLC–FT domino-like effect unfolds in slow motion over 5–6 months in both *E. japonicum* (Fig 2A–2C) and *A.halleri* (Fig 2D–2F), revealing features that are difficult to capture in short laboratory assays. First, *FLC* began to decline before *VIN3* expression peaked—late October in *E. japonicum* and late November in *A.halleri*—suggesting that an early, VIN3-independent phase of *FLC* repression, supported by genetic studies in *A. thaliana* [39,43], may already have been present in the perennial ancestor. During *FLC* dial-up, both species showed two transcriptional signatures as previously reported in *A.halleri* [22,24,34], indicating that these behaviours are conserved across species in the field. In spring, *FLC* and *VIN3* levels correlated inversely, a pattern not captured in laboratory studies where abrupt cold-to-warm shifts rapidly extinguish *VIN3* expression [13]. Moreover, *VIN3* reached its seasonal minimum before *FLC* attained its maximum, raising the possibility that the absence of *VIN3* is a permissive condition for re-establishing high *FLC* levels. Finally, *FLC* dial-up kinetics differed between species and were mirrored in *FT* peak shapes: in *E. japonicum*, the rapid *FLC* dial-up coincided with a narrower *FT* peak, whereas in *A.halleri* the slower *FLC* dial-up corresponded to a broader *FT* peak. This could be consistent with a FT-FLC feedback loop evidenced in the winter annual life history [44,45].

The conservation of the VIN3–FLC–FT domino-like sequence between *E. japonicum* and *A.halleri* suggests that the roles of *AhgFLC* and *AhgFT* may not be as tightly linked to reversion and flowering transitions, unique to the life history of *A.halleri*, as previously proposed [21,34] and also recently reported [46]. The mechanistic or biological basis within the VIN3–FLC–FT regulatory module awaits genetic analysis in their respective perennial life histories, which lie beyond the scope of this study. Instead, we regard the conserved features of the VIN3–FLC–FT network as the empirical basis for applying a more generic comparative measure—the thermal memory interval—introduced in the next and the last sections.

### Thermal memory interval adds predictive power to chromatin and expression features of spring and autumn *FLC* states

Previous work established that *AhgFLC* expression follows a seasonal cycle characterized by chromatin-based stability during both *AhgFLC* dial-down and *AhgFLC* dial-up, while these phases remain sensitive to the changing trend of temperatures in autumn and spring [7,22]. First, we addressed whether this feature is conserved in the distantly related perennial *E. japonicum*.

**Fig 3:**
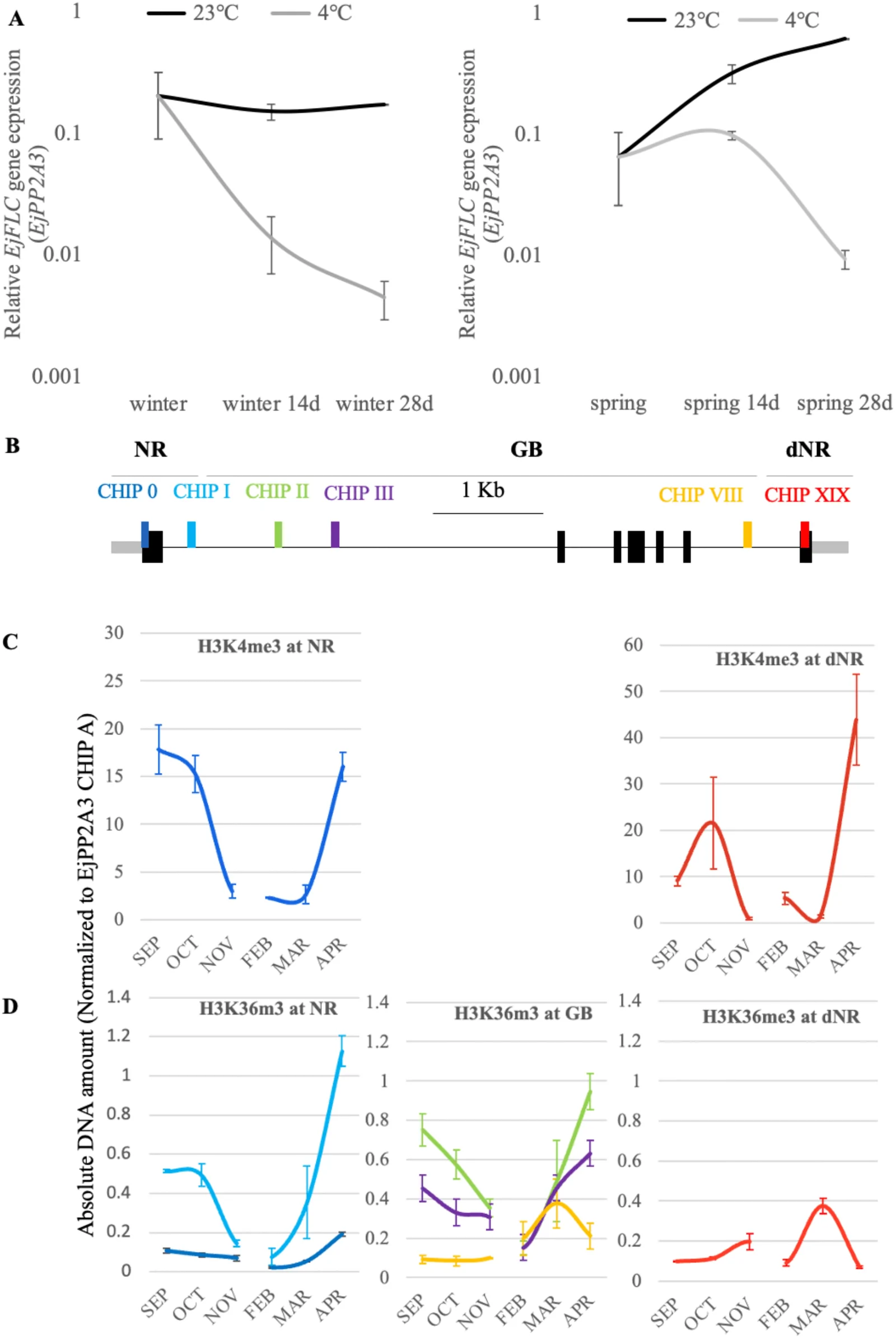
Asymmetry of *FLC* dialing states at gene expression and chromatin level in *Eutrema japonicum.* **(**A) Distinct transcription states *EjFLC* at onset of winter and spring. Average of three biological replicates of mRNA quantification of *EjFLC*, as indicated on the vertical axis, using 𝜟𝜟CT method and *EjPP2A3* as an internal control. The scale for each gene was chosen to maintain consistency throughout the manuscript. Lines represent smooth curves. Plants were transferred from Ikawa on 17 December 2018, ”winter”, and 18 March 2019, “spring”. (B) Distribution of ChIP amplicons relative to the *EjFLC* with untranslated regions (grey boxes), introns (lines) and exons (black). CHIP 0 and I are in the Nucleation Region (“NR”), CHIP II, III, VIII in the gene body (“GB”) and CHIP IX in the distal Nucleation Region (“dNR”). The three chromatin domains are approximated by the grey lines on the top. The genomic features, but not the ChIP amplicons, are drawn to scale. (C) Absolute quantification of H3K4me3 at regions NR (left) and dNR (right), normalized to EjPP2A3 CHIP A. (D) Absolute quantification of H3K36me3 along all regions, normalized to EjPP2A3 CHIP A. Colour code is the same in B-D.

In both species, *FLC* expression reaches mid-intermediate levels at two timepoints per year: once during *FLC* dial-down in early winter and again during *FLC* dial-up in mid-spring (Fig 2B, 2E). We selected these matched expression timepoints to test their temperature sensitivity in *E. japonicum*. Transfer experiments from natural conditions to two contrasting constant temperatures (4 °C and 23 °C) showed that temperature sensitivity differs seasonally (Fig 3A). In winter, *EjFLC* expression remained stable for four weeks following transfer to 23 °C, while in spring, the same transfer led to a gradual ~10-fold increase. Conversely, exposure to 4 °C caused a rapid *EjFLC* decline in winter-consistent with classical vernalization responsiveness-but had no effect in spring until four weeks later. These results mirror what was previously observed in *A.halleri* [21,22], confirming that the *FLC* dial-up and *FLC* dial-down phases are distinct in their responsiveness to temperature, even when transcript levels are matched. This represents an epigenetic hallmark: the same DNA sequence can give rise to different transcriptional outcomes depending on prior history. The differential behavior of these dialling states prompted us to verify whether corresponding distinct chromatin dynamics are conserved in *E. japonicum*.

To address this, we mapped selected chromatin dynamics at *EjFLC* (Fig 3B–3D). We note that the timepoints for ChIP assays in Fig 3 correspond with those for gene expression analysis in Fig 5 for September, to November and February to April (also indicated in S1 Table). We designed oligonucleotide probes targeting three regions previously defined in *AhgFLC*: the nucleation region (NR), gene body (GB), and distal nucleation region (dNR) (Fig 3B). Using ChIP-qPCR, we quantified active chromatin marks—H3K4me3 and H3K36me3—in the months preceeding the mid-intermediate FLC levels, i.e., during late autumn and late winter. At the NR (CHIP 0, I), levels of H3K4me3 and H3K36me3 varied quantitatively with *EjFLC* expression (Fig 3C; Ikawa control in Fig 5C), consistent with a role in modulating sense transcription. However, dynamics at other domains were distinct. At the GB, CHIP II reflected NR-like behavior, but CHIP III and VIII did not. These sites showed no decline in H3K36me3 from October to November (despite ~15-fold transcriptional downregulation, Fig 5C) and no increase from March to April (during ~30-fold transcriptional upregulation, Fig 5C). At the dNR (CHIP IX), H3K36me3 levels increased during autumnal dial-down—opposite to transcription—and decreased in spring during dial-up (Figs 3C and 3D, 5C). This pattern may suggest the existence of an antisense-mediated regulatory mechanism (e.g., *COOLAIR*), which was previously proposed for *AhgFLC* [22],although the specific regulatory mechanisms underlying these dynamics in *E. japonicum* remain unresolved. These data indicate that, while the NR chromatin state aligns with expression, distal regions such as the GB and dNR maintain distinct histone modification patterns that are not transcription-coupled and exhibit seaonal variation. These distinct chromatin domains at *EjFLC* match the organizational logic observed at *AhgFLC* [22] thereby supporting conservation in *E. japonicum*. We note that, as ChIP amplicons were designed in regions of 100% sequence identity, the chromatin profiles in Fig 3 represent the combined signal from both *EjFLC* homeologues and therefore reflect their average epigenetic state; locus-specific differences cannot be excluded.

**Fig 4:**
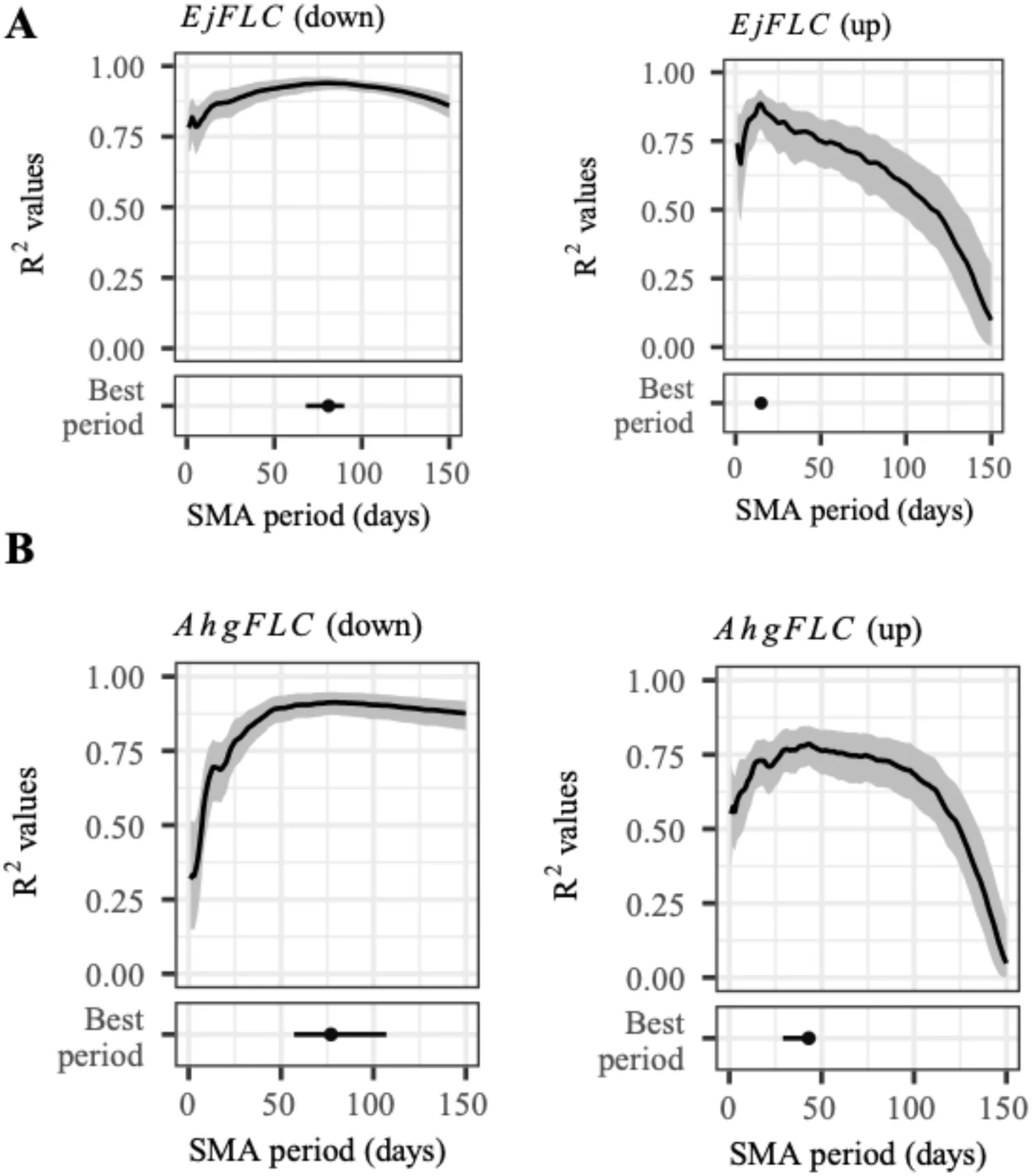
Linear regression analyses of *EjFLC* and *AhgFLC* mRNA levels against the simple moving average (SMA) of past temperature during dial-up and dial-down phases. (A, B) R^2^ values from linear regression using SMAs of daily mean temperature with different window lengths, separately for the *FLC* dial-up (right) and *FLC* dial-down (left) phases, for *EjFLC* (A) and *AhgFLC* (B). Lines and shaded areas represent the median and 95% confidence intervals of 1,000 bootstrap samples, respectively. The median and 95% confidence intervals of the best SMA period are shown below the R^2^ plots.

Next, we sought to distinguish the dialling states quantitatively by measuring the thermal memory interval of *FLC* expression—the time window of past temperature that best predicts mRNA levels— specifically during the dialling phases, using simple moving averages (SMAs) and linear regression (Fig 4; S4 Fig). During *FLC* dial-up (March–May), *FLC* integrated recent temperature changes over short intervals: 14–15 days in *E. japonicum* and 29–44 days in *A.halleri* (Fig 4A, 4B right). In contrast, during *FLC* dial-down (September–February in *E. japonicum*; November–February in *A.halleri*), memory intervals were substantially longer: 68–90 days and 57–107 days, respectively (Fig 4A, 4B left). This reveals a new feature of FLC regulation—season-dependent memory length—which is conserved across species. Importantly, the distinction in TMIs between *FLC* dial-up and *FLC* dial-down recapitulates mechanistic differences previously attributed to *cis*-based chromatin and *trans*-acting factors [7]. Thus, TMIs provide a quantitative parameter of time-integrative memory length that aligns with known regulatory mechanisms but can be derived from expression and environmental data alone. In this way, TMIs extend mechanistic insights into a generalizable and comparative measure of time-integrative memory across contexts.

### VIN3–FLC–FT network maintains flexibility and robustness while encoding distributed memory

**Fig 5:**
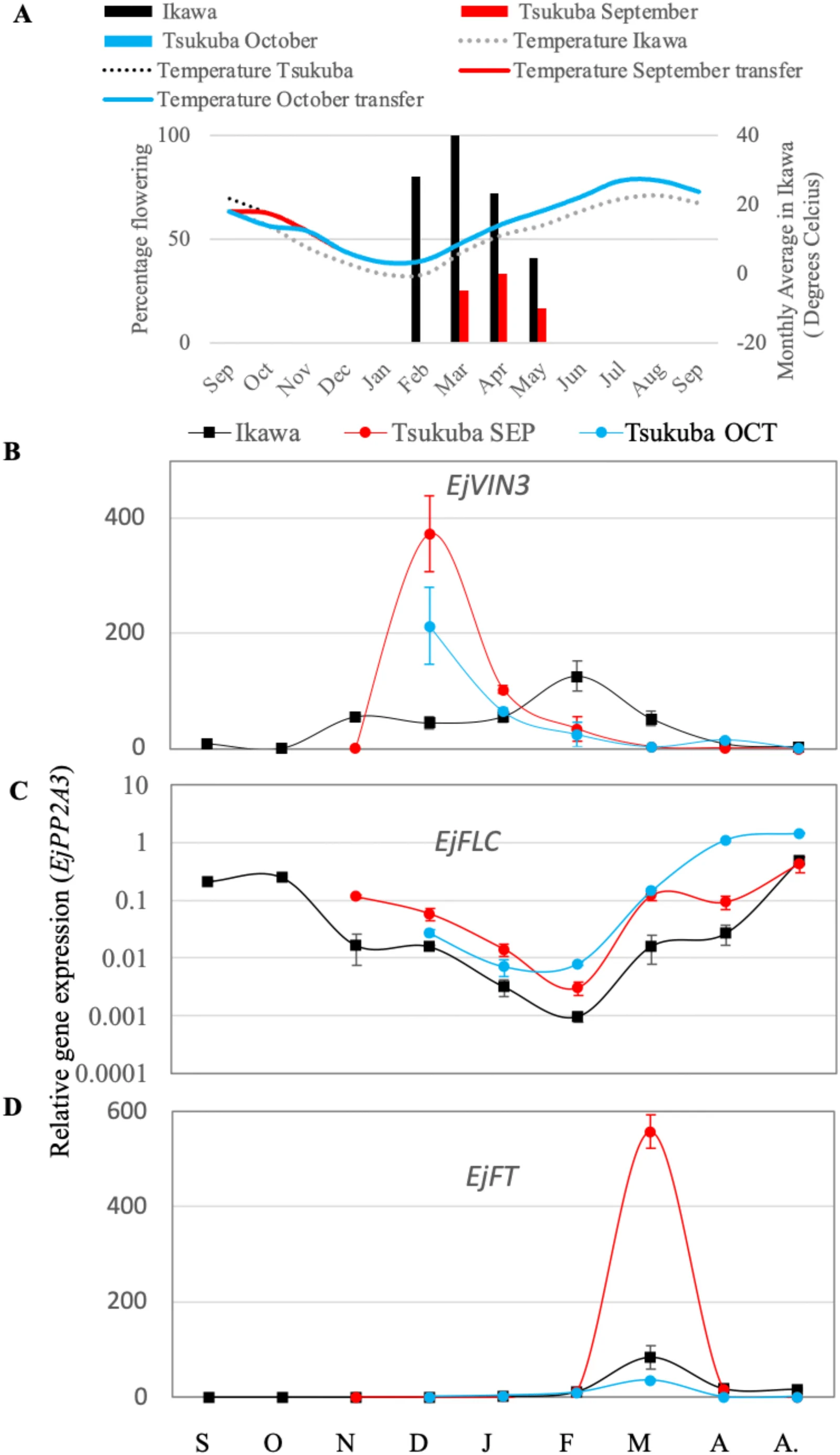
*E. japonicum* *VIN3*-*FLC*-*FT* module dynamics at the native and a remote site. (A) Flowering time against monthly average of temperature in transfer experiments from Ikawa to Tsukuba performed in September and October. (B-D) Gene expression analysis of *EjVIN3*, *EjFLC* and *EjFT* in Tsukuba transfer experiments against the Ikawa control. Average of three biological replicates of mRNA quantification for all genes, as indicated on the vertical axis, using 𝜟𝜟CT method and *EjPP2A3* as an internal control. The scale for each gene was chosen to maintain consistency throughout the manuscript. Lines represent smooth curves. S, 28 September 2021; O, 26 October 2021; N, 25 November 2021; D, 28 December 2021; J, January 30 2022; F, 25 February 2022; M, 15 March 2022; A, 4 April 2022, A., 25 April 2022.

To examine how the VIN3–FLC–FT network responds to environmental conditions different from its native site and to assess the ability of this network to respond to long-past environmental histories, we conducted transfer experiments from the native site in Ikawa to a warmer location in Tsukuba (Fig 5A). Average hourly temperatures in Tsukuba were at least 4 °C higher than in Ikawa for most of the year (S8 Fig), a temperature difference previously shown to influence vernalization dynamics and flowering phenology in *A.halleri* [34]. Flowering time and gene expression were monitored following transfers at the onset of *EjFLC* dial-down in September and October, over six to seven months. Flowering differed from native conditions in both transfers: in the September transfer, 70% of plants failed to flower and 30% flowered late, whereas in the October transfer, no plants flowered at all (Fig 5A). These phenotypes may be consistent with elevated *EjFLC* (both transfers) or reduced *EjFT* expression (October transfer) or both (September transfer), given that *EjFLC* inhibit flowering (Fig 1D) whereas *EjFT* promotes it [35]. Also, flowering phenotypes of our transfer experiments are consistent with warming studies in *A.halleri* [34,47].

Despite aberrant flowering, the VIN3–FLC–FT domino-like effect remained recognizable in the monitored cellular lineage, indicating that this regulatory sequence can operate beyond, and is not entirely coupled to, the floral transition. The characteristic four-quadrant expression pattern of *EjFLC* remained recognizable (Fig 5B–5D). Each gene displayed distinct response dynamics relative to the environment difference between the two transfers. Notably, *EjVIN3* diverged 2–3 months after transfer. In addition, *VIN3* exhibited earlier induction in the transfer experiments than what is typically observed in native populations as well as divergent expression levels between the two transfer groups in December (Fig 5B). This divergence may reflect exposure to non-native environmental conditions, including differences in both temperature and light between the forest understory, canopy-filtered environment at Ikawa and the open-field, full-sun conditions at Tsukuba, as well as may minor variation in sampling time within the defined collection windows, given that *VIN3* is regulated by the circadian clock [48]. In contrast, *EjFLC* diverged throughout the entire interval analyzed (Fig 5C). Strikingly, *EjFT* “memorized” the autumn environmental variation until spring, as indicated by the of change in amplitude of *EjFT* expression in March (Fig 5D). These observations indicate that individual network components can respond to environmental changes over different timescales while maintaining the overall regulatory framework, demonstrating the flexibility and robustness of the network. The differences in response timing among *EjVIN3*, *EjFLC,* and *EjFT* suggest that environmental history is distributed across the network.

### Thermal memory intervals vary with genes and species and reveal distributed effects

**Fig 6:**
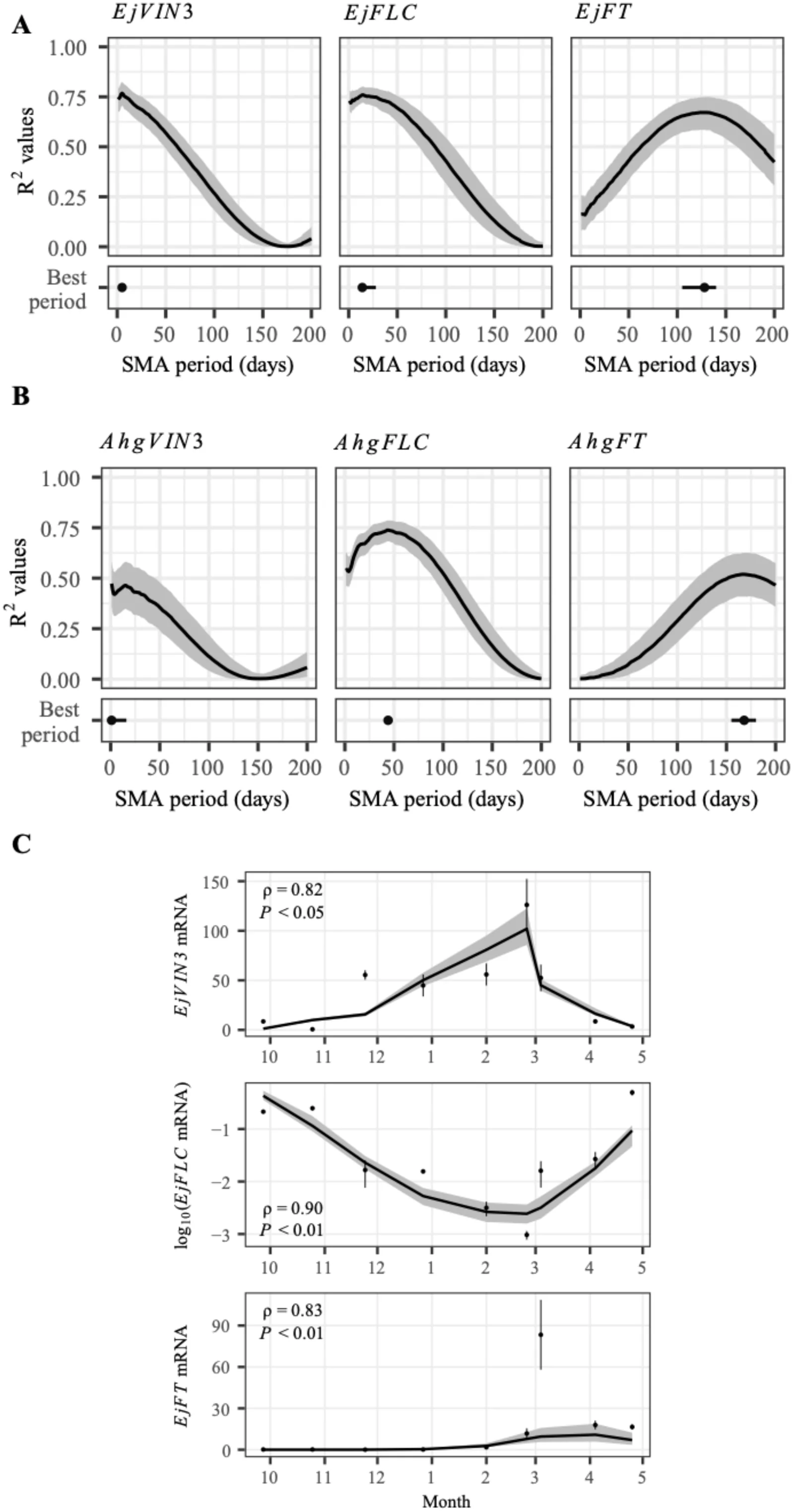
Linear regression analyses and model-based prediction of mRNA dynamics using the simple moving average (SMA) of past temperature. (A, B) R^2^ values from linear regression of mRNA levels against SMAs of daily mean temperature with different window lengths, based on the two-year mRNA dynamics of *Eutrema japonicum* (A) and *Arabidopsis halleri* (B) shown in Fig 2. (C) Model predictions of mRNA dynamics in *E. japonicum* from 28 September 2021 to 25 April 2022 (Fig 5), using the regression model in (A) with the best SMA period (highest R^2^). Spearman’s correlation coefficients (ρ) between observed and predicted values and their *P* values are shown. In (A-C), lines and shaded areas represent the median and 95% confidence intervals of 1,000 bootstrap samples, respectively. In (A, B), the median and 95% confidence intervals of the best SMA period are shown below the R^2^ plots.

Seasonal expression of *VIN3*, *FLC*, and *FT* showed broadly similar patterns across divergent species (Fig 2), while their response timescales differed under non-native conditions (Fig 5). Motivated by the need for a standardized metric, we sought to quantify these similarities and differences further. While the analysis in Fig 4 focused on distinguishing the distinct kinetic rules governing the two *FLC* dialling phases, we next sought to calculate the TMIs of *VIN3*, *FLC*, and *FT* across the entire year in both species (Fig 6A, 6B). To achieve this, we performed linear regression analyses relating gene expression to simple moving averages (SMAs) of daily mean temperature across multiple time windows (Fig 6A, 6B; S5–S7 Figs) and across the full two-year censuses. For each gene, the SMA window that best explained transcript abundance differed between genes and species (Fig 6A, 6B). However, a consistent trend emerged across the two species: *VIN3* integrated temperature over shorter windows (4–6 days for *EjVIN3*, 1–16 days for *AhgVIN3*), *FLC* over intermediate windows (13–28 days for *EjFLC*, 43–48 days for *AhgFLC*), and *FT* over longer windows (104–150 days for *EjFT*, 150 days for *AhgFT*). This indicates that TMIs are distributed along the regulatory cascade, possibly reflecting both each gene’s intrinsic regulatory features and contributions from upstream components. For example, *FLC*’s TMI, largely determined by its *cis* chromatin switch [7,8,10,22] could extend *FT*’s TMI.

Our results provide the first estimates of TMIs for *VIN3* and *FT* in two perennial species, as well as extend the time-integrative concept initially identified at *FLC* in *A. halleri*. Our data also independently reproduced the estimates of Aikawa et al. (2010) using a different field census from 2012–2014, yielding a nearly identical TMI range of 43–48 days. These results indicate that TMI is consistent across separate field studies spanning six years. We reasoned that if temperature history is the primary and consistent predictor of transcriptional states across the vernalization cascade, a regression model derived from our initial *E. japonicum* census (2016–2018) should reliably forecast mRNA dynamics in an independent year (2021–2022). Indeed, the regression model developed in Fig 6A accurately predicted the seasonal dynamics observed in the independent year (Fig 5B) with high accuracy (Spearman’s ρ > 0.8; Fig 6C). The critical transition points for *EjFLC*, *EjVIN3*, and *EjFT* were well captured using past temperature data alone, with the exception of the full amplitude of the *EjFT* peak in March. While seasonal timing was accurately predicted, the amplitude of the *EjFT* peak was not, which is consistent with the burst-like transcriptional behaviour of *EjFT* observed in the census data (Fig 2C). Overall, these findings demonstrate that the integrated history of past temperatures is sufficient to explain seasonal phase switching at *VIN3*, *FLC*, and *FT* in nature, and once again that TMI represents a consistent metric of integrative thermal memory. While these genes are also subject to chromatin regulation [49,50], their TMIs could be resolved without prior mechanistic knowledge, illustrating how this approach might be applicable to other genes and contexts. FLC has long been recognized as a striking case of time-integrative memory, integrating past temperatures over ~6 weeks [21]. Here, we show that *FT* integrates temperature over even longer periods, up to ~21 weeks, revealing that thermal memory can operate across broader timescales, and further highlighting the potential for TMIs to uncover similar patterns in other genes and species. The conservation of this progressive pattern across two divergent *Brassicaceae* suggests shared, modular principles for encoding seasonal environmental history that are independent of the perennial type of life history.

While TMIs provide a powerful and generalizable metric for time-integrative memory, their limitations should be acknowledged. Calculating TMIs requires dense, high-resolution gene expression and environmental data, such as the biweekly census collected over two years in this study. TMIs capture memory timescales but do not reveal underlying molecular mechanisms, including *cis*-encoded chromatin states, trans-acting regulation, or feedback loops. Long intervals—such as those observed for FT—may reflect cumulative contributions from upstream components rather than intrinsic memory at that gene. Nevertheless, building on the distributed memory observed in the *VIN3 – FLC–FT* network, TMIs offer a versatile framework to explore how broadly time-integrative memory operates across other genes, species, and environmental contexts. Moreover, high-resolution gene expression data can be analysed against additional variables—such as anatomical or physiological traits [51] or functional readouts such as photosynthetic efficiency [52]—extending the utility of TMIs to capture how genes integrate fluctuating signals beyond temperature.

## Conclusions

Comparative field-based studies of *A.halleri* and *E. japonicum* revealed that perennial *Brassicaceae* with divergent life histories nonetheless share core features of *FLC* and the VIN3–FLC–FT regulatory module. By relating gene expression to past temperature, we applied the Thermal Memory Interval (TMI) as a transferable, quantitative measure of time-integrative memory. Calculating TMIs required a high-resolution census of gene expression over two years under natural conditions, combined with meteorological data, enabling the capture of seasonal transitions, the distinction of autumn and spring *FLC* phases, and the quantification of distributed information flow across *VIN3*, *FLC*, and *FT*. Importantly, TMIs provide a unified readout of time-integrative memory without prior mechanistic knowledge. Because the TMI can be derived directly from high-resolution expression data alongside other dense datasets—such as environmental, anatomical, or physiological measurements—this approach is broadly generalizable beyond the three genes and single environmental variable examined here, offering a comparative framework for studying cellular memory across genes, species, and contexts.

## Supporting information

S1 Fig Two distinct perennial life histories. A-C. **Atypical perennial life history of** ***Arabidospis halleri gemmifera.*** Plants grown in a meshed glasshouse with windows were open to maintain near outdoor temperature in Tsukuba from September 2023 to May 2024 were pressed at different stages of development. (A) Mature rosette (October) (B). Full flowering stage (early March). Note that the number of inflorescence stems (red arrows) is about the same as the number of rosette leaves. Since each rosette leaf is part of a repeating metamer composed of leaf, internode and subtended meristem, this implies that all vegetative meristems were induced to flower (C) Full reversion stage (early May), at the time all meristems, apical and axillary, have reverted into vegetative growth and developed into aerial rosettes (blue arrows). (D). **Flowering stage in *Eutrema japonicum***. Representative plants from April 2022 from the flowering subset of the Ikawa to Tsukuba September transfer experiment (also see Fig 5, S8 Fig).
(TIFF)

S2 Fig Sequence analysis of *FLC* coding regions in *Eutrema japonicum.* (A) DNA sequence polymorphisms and resulting amino acid differences between two non-identical (aminoacid level) identified clones (*1* and *2)* and *EjFLC-A* and *EjFLC-B.* Numbers indicate position of the base pair from ATG codon. (B) Multiple sequence alignment between *AtFLC*, *EjFLC-A*, *EjFLC-B*. Amino acid differences between two species are in red. Arrow indicate amino acid difference between *EjFLC-A* and *EjFLC-B*.
(TIFF)

S3 Fig *EjFLC* genes are not differentially expressed throughout the year. Percentage of cDNA clones for each *EjFLC* gene, curated based on the presence of the six basepair substitutions from chromatographs from March 8, 2017, May 24, 2017, June 14, 2017, July 12, 2017, September 21, 2016, and December 14 2016. The number of clones sequenced from each sample is indicated in brackets.
(TIFF)

S4 Fig Linear regression of *EjFLC* (A, C) and *AhgFLC* (B, D) gene expression levels on simple moving average of past temperature with different window lengths during *FLC* dial up (A, C) and *FLC* dial down (B, D). Gene expression level relative to *EjPP2A3* or *AhgPP2A3* was plotted against the simple moving average (SMA) of past temperature with window length of 1day, 20, 40, 80 and 150 days for both genes. The time points used for the analyses can be found under “Statistical modelling” of the “Materials and Methods” section. Coefficient of determination (R^2^) is shown within the plots.
(TIFF)

S5 Fig Overlay between *FLC* expression and simple moving average of past temperature with different window lengths over 2 years in *Eutrema japonicum* (A) and *Arabidopsis halleri gemmifera* (B). The simple moving average (SMA) of past temperature with window length of 1 day, 20, 40, 80 and 150 days during the two years are shown in different colors, together with *EjFLC* (A) and *AhgFLC* (B) expression.
(TIFF)

S6 Fig Linear regression of *EjVIN3*, *EjFLC*, *EjFT* expression levels on simple moving average of past temperature with different window lengths. Gene expression level relative to *EjPP2A3* was plotted against the simple moving average (SMA) of past temperature with window Length of 1day, 10, 20, 30, 40 days for *EjVIN3* (A) and *EjFLC* (B) and of 1 day, 20, 40, 80, 150 days for *EjFT* (C). Coefficient of determination (R^2^) is shown within the plots.
(TIFF)

S7 Fig Linear regression of *AhgVIN3*, *AhgFLC*, *AhgFT* expression levels on simple moving average of past temperature with different window lengths. Gene expression level (relative to *AhgPP2A3*) was plotted against the simple moving average (SMA) of past temperature with window length of 1day, 10, 20, 30, 40 days for *AhgVIN3* (A) and *AhgFLC* (B) and of 1 day, 20, 40, 80, 150 days for *AhgFT* (C). Coefficient of determination (R^2^) is shown within the plots.
(TIFF)

S8 Fig Examples of *E. japonicum* plants from Ikawa to Tsukuba transplant experiments. (A) The twelve plants sampled in Ikawa on September 28 2021 on arrival in the lab in Tsukuba (one day after sampling in Ikawa) (B-C) Plants growing outdoor in Tsukuba, at the time of sampling in November and December.
(TIFF)

S9 Fig Annual hourly temperatures at the site of origin (Ikawa) and remote site (Tsukuba) of the transfer experiment. Hourly temperatures in Ikawa and Tsukuba and the difference in temperature over the interval 20 September 2021 to 20 August 2022.
(TIFF)

S10 Fig All supporting data. (XLSX)

S1 Table *E. japonicum* sample repository. (DOCX)

S2 Table List of oligonucleotides use in this study. (DOCX)
