# Supplementary material for: Conserved gene- and network-level thermal memory intervals in two divergent perennial crucifers in nature
Source: PLoS One. 2026 Aug 19;21(8):e0336733. doi: 10.1371/journal.pone.0336733 (PMC13489412; doi:10.1371/journal.pone.0336733)
Supplement: Supplementary file 1 [file pone.0336733.s011.docx]

**Table S1. Wasabi sample repository**

|  |  | Two year census IKAWA Mountain Center |  |  |  |
| --- | --- | --- | --- | --- | --- |
|  | Year | Day | Assay | Manuscript Figures |  |
|  | 2016 | 24-Aug-16 | qRT-PCR | 2,4,6,S4,S5,S6 |  |
|  |  | 7-Sep-16 | qRT-PCR | 2,4,6,S4,S5,S6 |  |
|  |  | 21-Sep-16 | qRT-PCR,EjFLC cDNA sequencing,cloning | 1E,2,4,6,S2,S3 |  |
|  |  | 5-Oct-16 | qRT-PCR | 2,4,6,S4,S5,S6 |  |
|  |  | 19-Oct-16 | qRT-PCR | 2,4,6,S4,S5,S6 |  |
|  |  | 2-Nov-16 | qRT-PCR | 2,4,6,S4,S5,S6 |  |
|  |  | 16-Nov-16 | qRT-PCR | 2,4,6,S4,S5,S6 |  |
|  |  | 30-Nov-16 | qRT-PCR | 2,4,6,S4,S5,S6 |  |
|  |  | 14-Dec-16 | qRT-PCR, EjFLC cDNA sequencing | 2,4,6,S2,S3 |  |
|  |  | 28-Dec-16 | qRT-PCR | 2,4,6,S4,S5,S6 |  |
|  | 2017 | 11-Jan-17 | qRT-PCR, | 2,4,6,S4,S5,S6 |  |
|  |  | 25-Jan-17 | qRT-PCR | 2,4,6,S4,S5,S6 |  |
|  |  | 8-Feb-17 | qRT-PCR | 2,4,6,S4,S5,S6 |  |
|  |  | 22-Feb-17 | qRT-PCR | 2,4,6,S4,S5,S6 |  |
|  |  | 8-Mar-17 | qRT-PCR,EjFLC cDNA sequencing | 2,4,6,S2,S3 |  |
|  |  | 22-Mar-17 | qRT-PCR | 2,4,6,S4,S5,S6 |  |
|  |  | 5-Apr-17 | qRT-PCR | 2,4,6,S4,S5,S6 |  |
|  |  | 19-Apr-17 | qRT-PCR | 2,4,6,S4,S5,S6 |  |
|  |  | 3-May-17 | qRT-PCR | 2,4,6,S4,S5,S6 |  |
|  |  | 10-May-17 |  | 2,4,6,S4,S5,S6 |  |
|  |  | 24-May-17 | qRT-PCR,EjFLC cDNA sequencing | 2,4,6,S2,S3 |  |
|  |  | 31-May-17 | qRT-PCR | 2,4,6,S4,S5,S6 |  |
|  |  | 14-Jun-17 | qRT-PCR,EjFLC cDNA sequencing | 2,4,6,S2,S3 |  |
|  |  | 28-Jun-17 | qRT-PCR | 2,4,6,S4,S5,S6 |  |
|  |  | 12-Jul-17 | qRT-PCR,EjFLC cDNA sequencing | 2,4,6,S2,S3 |  |
|  |  | 26-Jul-17 | qRT-PCR | 2,4,6,S4,S5,S6 |  |
|  |  | 9-Aug-17 | qRT-PCR | 2,4,6,S4,S5,S6 |  |
|  |  | 5-Sep-17 | qRT-PCR | 2,4,6,S4,S5,S6 |  |
|  |  | 20-Sep-17 | qRT-PCR | 2,4,6,S4,S5,S6 |  |
|  |  | 4-Oct-17 | qRT-PCR | 2,4,6,S4,S5,S6 |  |
|  |  | 18-Oct-17 | qRT-PCR | 2,4,6,S4,S5,S6 |  |
|  |  | 1-Nov-17 | qRT-PCR | 2,4,6,S4,S5,S6 |  |
|  |  | 15-Nov-17 | qRT-PCR | 2,4,6,S4,S5,S6 |  |
|  |  | 29-Nov-17 | qRT-PCR | 2,4,6,S4,S5,S6 |  |
|  |  | 13-Dec-17 | qRT-PCR | 2,4,6,S4,S5,S6 |  |
|  |  | 27-Dec-17 | qRT-PCR | 2,4,6,S4,S5,S6 |  |
|  | 2018 | 10-Jan-18 | qRT-PCR | 2,4,6,S4,S5,S6 |  |
|  |  | 24-Jan-18 | qRT-PCR | 2,4,6,S4,S5,S6 |  |
|  |  | 7-Feb-18 | qRT-PCR | 2,4,6,S4,S5,S6 |  |
|  |  | 14-Feb-18 | qRT-PCR | 2,4,6,S4,S5,S6 |  |
|  |  | 21-Feb-18 | qRT-PCR | 2,4,6,S4,S5,S6 |  |
|  |  | 7-Mar-18 | qRT-PCR | 2,4,6,S4,S5,S6 |  |
|  |  | 14-Mar-18 | qRT-PCR | 2,4,6,S4,S5,S6 |  |
|  |  | 28-Mar-18 | qRT-PCR | 2,4,6,S4,S5,S6 |  |
|  |  | 11-Apr-18 | qRT-PCR | 2,4,6,S4,S5,S6 |  |
|  |  | 25-Apr-18 | qRT-PCR | 2,4,6,S4,S5,S6 |  |
|  |  | 9-May-18 | qRT-PCR | 2,4,6,S4,S5,S6 |  |
|  |  | 23-May-18 | qRT-PCR | 2,4,6,S4,S5,S6 |  |
|  |  | 7-Jun-18 | qRT-PCR | 2,4,6,S4,S5,S6 |  |
|  |  | 4-Jul-18 | qRT-PCR | 2,4,6,S4,S5,S6 |  |
|  |  | 11-Jul-18 | qRT-PCR | 2,4,6,S4,S5,S6 |  |
|  |  | 25-Jul-18 | qRT-PCR | 2,4,6,S4,S5,S6 |  |
|  |  | 8-Aug-18 | qRT-PCR | 2,4,6,S4,S5,S6 |  |
|  |  | 22-Aug-18 | qRT-PCR | 2,4,6,S4,S5,S6 |  |
|  |  | 5-Sep-18 | qRT-PCR | 2,4,6,S4,S5,S6 |  |
|  |  | 12-Sep-18 | qRT-PCR, | 2,4,6,S2 |  |
|  |  | 19-Sep-18 | qRT-PCR | 2,4,6,S4,S5,S6 |  |
|  |  | 17-Dec-18 | qRT-PCR, temperature transfer | 3A |  |
|  | 2019 | 18-Mar-19 | qRT-PCR, temperature transfer | 3A |  |
|  |  |  |  |  |  |
|  |  |  |  |  |  |
|  |  |  |  |  |  |
|  |  |  |  |  |  |

| Mid-intermediate temperature transfers | | |  |  |
| --- | --- | --- | --- | --- |
| University of Tsukuba Laboratory | | |  |  |
|  |  |  |  |  |
| Year | Day | Assay | Manuscript Figures | Sample name |
| 2018 | 19-Dec-18 | q-RTPCR | 3A | winter |
|  |  | q-RTPCR | 3A | winter 14d |
|  |  | q-RTPCR | 3A | winter 28 |
|  |  |  |  |  |
| 2019 | 18-Mar-19 | q-RTPCR | 3A | spring |
|  |  | q-RTPCR | 3A | spring 14d |
|  |  | q-RTPCR | 3A | spring 28d |

| IKAWA Mountain Center | | |  |  |
| --- | --- | --- | --- | --- |
|  |  | Transfer from Ikawa Mountain Center to Tsukuba outdoor |  |  |
|  |  |  |  |  |
| Year | Day | Assay | Manuscript Figures | Sample name |
| 2021 | 27-Sep-21 | qRT-PCR, phenotyping,transfer exp, ChIP q-PCR | 1D,3,5,6C | SEP/S |
|  | 25-Oct-21 | qRT-PCR, phenotyping,transfer exp, ChIP q-PCR | 1D,3,5, 6C | OCT/O |
|  | 24-Nov-21 | qRT-PCR, phenotyping, ChIP q-PCR | 1D,3,5, 6C | NOV/N |
|  | 27-Dec-21 | qRT-PCR, phenotyping | 1D,3,5, 6C | DEC/D |
| 2022 | 31-Jan-22 | qRT-PCR, phenotyping | 1D,3,5, 6C | JAN/J |
|  | 24-Feb-22 | qRT-PCR, phenotyping, ChIP q-PCR | 1D,3,5, 6C | FEB/F |
|  | 14-Mar-22 | qRT-PCR, phenotyping, ChIP q-PCR | 1D,3,5, 6C | MAY/M |
|  | 4-Apr-22 | qRT-PCR, phenotyping | 1D,3,5, 6C | APR/A |
|  | 25-Apr-22 | qRT-PCR, phenotyping, ChIP q-PCR | 1D,3,5, 6C | APR/A. |
|  | 16-May-22 | qRT-PCR, phenotyping | 1D,3,5 | MAY |
|  | 13-Jun-22 | phenotyping | 1D,5 | JUN |
|  | 11-Jul-22 | phenotyping | 1D,5 | JUL |
|  | 10-Aug-22 | phenotyping | 1D,5 | AUG |
