# Supplementary material for: Conserved gene- and network-level thermal memory intervals in two divergent perennial crucifers in nature
Source: PLoS One. 2026 Aug 19;21(8):e0336733. doi: 10.1371/journal.pone.0336733 (PMC13489412; doi:10.1371/journal.pone.0336733)
Supplement: Supplementary file 2 [file pone.0336733.s012.docx]

**Table S2. List of oligonucleotides used in this study.**

| Assay | Oligo Name | Oligo sequence (5' to 3') | |  |  |  |
| --- | --- | --- | --- | --- | --- | --- |
| Eutrema japonicum |  |  |  |  |  |  |
| CLONING, | attb1EjFLCs cDNAfull | GGGGACAAGTTTGTACAAAAAAGCAGGCTTCATGGGAAGAAAAAAACTA | | | | |
| SEQUENCING | attb2EjFLCs cDNAfull | GGGGACCACTTTGTACAAGAAAGCTGGGTTGCTAATTAAGCAGTGGGAGAGTT | | | | |
|  | attb2genomicFLC 5.9 kb | GGGGACCACTTTGTACAAGAAAGCTGGGTGTTCTGACTTTTACCCGTGC | | | | |
| QRT-PCR | EjFLC cDNA F | GAGGATCACCTGGAGACTGC | | |  |  |
|  | EjFLC cDNA R | CTTCTGCTCCCACATGATGA | | |  |  |
|  | EjVIN3 cDNA F | TGTGGGTCCAGAAAACGTAGAT | | |  |  |
|  | EjVIN3 cDNA R | ATCTCATTTCGGGTCCAAGAGT | | |  |  |
|  | EjFT cDNA F | GGTGGGGAGAGTTGTTACCG | | |  |  |
|  | EjFT cDNA R | AGGTGAGGGTTGCTAGGACT | | |  |  |
|  | EjPP2A3 cDNA F | GTATGCACATGTTTTGCTTCCAC | | |  |  |
|  | EjPP2A3 cDNA R | CAACCAAGTCATTCTCCCTCATC | | |  |  |
| Q-CHIP PCR | EjFLC CHIP 0 F | AAATAAATACTCCGGCGACTTGAAC | | |  |  |
|  | EjFLC CHIP 0 R | CGTCGTTTGGAGAAGGTGAC | | |  |  |
|  | EjFLC CHIP I F | TTTCAGGCAGAACAATGTCG | | |  |  |
|  | EjFLC CHIP I R | AAGATCTTGAAGGAACATGC | | |  |  |
|  | EjFLC CHIP II F | TTTGAATTTTGGTAGCTTTAAGAAAAC | | |  |  |
|  | EjFLC CHIP II R | GACTAAAAATAGACCAATTGATGCAC | | |  |  |
|  | EjFLC CHIP III F | GCACATACAACACTTTGTTCC | | |  |  |
|  | EjFLC CHIP III R | GGTTGTCACTTTCCGAGAGG | | |  |  |
|  | EjFLC CHIP VIII F | AAGGCAGAAGTTGAAGATGAAA | | |  |  |
|  | EjFLC CHIP VIII R | ACAAAACAAAACCGGTTCCA | | |  |  |
|  | EjFLC CHIP IX F | TGATGTCGGATATTTGTCTAGGTG | | |  |  |
|  | EjFLC CHIP IX R | ATGGAGAAGAATCATCATGTGG | | |  |  |
|  | EjPP2A3 CHIP A F | GCTTTCTACGATTGCCCGAG | | |  |  |
|  | EjPP2A3 CHIP A R | GCATACTCAACGCCTCCAAC | | |  |  |
| Arabidoposis halleri subsp gemmiefa |  |  |  |  |  |  |
| QRT-PCR | AhgFLC cDNA F | CGGCGGTTAAAATCAAAATC | | |  |  |
|  | AhgFLC cDNA R | CAAACGCTCGCCCTTATC | |  |  |  |
|  | AhgVIN3 cDNA F | CTCGGATGCTGGAGGAAACA | | |  |  |
|  | AhgVIN3 cDNA R | TTCTTCACCGCCTCATCCAT | | |  |  |
|  | AhgFT cDNA F | TGGTGGATCCAGATGTTCCTAGT | | |  |  |
|  | AhgFT cDNA R | TCACCAACCAATGGAGATATTCTC | | |  |  |
|  | AhgPP2A3 cDNA F | GTATGCACATGTTTTGCTTCCAC | | |  |  |
|  | AhgPP2A3 cDNA R | CAAACGCTCGCCCTTATC | |  |  |  |
