# Supplementary material for: Conserved gene- and network-level thermal memory intervals in two divergent perennial crucifers in nature
Source: PLoS One. 2026 Aug 19;21(8):e0336733. doi: 10.1371/journal.pone.0336733 (PMC13489412; doi:10.1371/journal.pone.0336733)
Supplement: Supplementary file 3 [file pone.0336733.s014.docx]

**View Letter**

| **Date:** | Mar 17 2026 12:46AM |
| --- | --- |
| **To:** | "Diana Mihaela Mihaela Buzas" buzas.mihaela.ka@u.tsukuba.ac.jp |
| **From:** | "PLOS ONE" plosone@plos.org |
| **Subject:** | PLOS ONE Decision: Revision required [PONE-D-25-58585] |

PONE-D-25-58585

Conserved gene- and network-level thermal memory intervals in two divergent perennial crucifers in nature

PLOS One

Dear Dr. Buzas,

Thank you for submitting your manuscript to PLOS ONE. After careful consideration, we feel that it has merit but does not fully meet PLOS ONE’s publication criteria as it currently stands. Therefore, we invite you to submit a revised version of the manuscript that addresses the points raised during the review process.

The manuscript would benefit from a clearer conceptual framework that explicitly addresses TMI and its role within the proposed model and extending it to E. japonicum. Background information on flowering genes; FLC, FT and VIN will be useful to readers. I agree with both reviewers' comments. Please go through, make corrections and address each point

Please submit your revised manuscript by May 01 2026 11:59PM. If you will need more time than this to complete your revisions, please reply to this message or contact the journal office at [plosone@plos.org](mailto:plosone@plos.org). When you're ready to submit your revision, log on to https://www.editorialmanager.com/pone/ and select the 'Submissions Needing Revision' folder to locate your manuscript file.

Please include the following items when submitting your revised manuscript:

- A letter that responds to each point raised by the academic editor and reviewer(s). You should upload this letter as a separate file labeled 'Response to Reviewers'.
- A marked-up copy of your manuscript that highlights changes made to the original version. You should upload this as a separate file labeled 'Revised Manuscript with Track Changes'.
- An unmarked version of your revised paper without tracked changes. You should upload this as a separate file labeled 'Manuscript'.

If you would like to make changes to your financial disclosure, please include your updated statement in your cover letter. Guidelines for resubmitting your figure files are available below the reviewer comments at the end of this letter.

If applicable, we recommend that you deposit your laboratory protocols in protocols.io to enhance the reproducibility of your results. Protocols.io assigns your protocol its own identifier (DOI) so that it can be cited independently in the future. For instructions see: <https://journals.plos.org/plosone/s/submission-guidelines#loc-laboratory-protocols>. Additionally, PLOS ONE offers an option for publishing peer-reviewed Lab Protocol articles, which describe protocols hosted on protocols.io. Read more information on sharing protocols at <https://plos.org/protocols?utm_medium=editorial-email&utm_source=authorletters&utm_campaign=protocols>.

We look forward to receiving your revised manuscript.

Kind regards,

Harsh Raman, Ph.D
Academic Editor
PLOS One

Journal Requirements:

When submitting your revision, we need you to address these additional requirements.

1.Please ensure that your manuscript meets PLOS ONE's style requirements, including those for file naming. The PLOS ONE style templates can be found at

https://journals.plos.org/plosone/s/file?id=wjVg/PLOSOne_formatting_sample_main_body.pdf and

<https://journals.plos.org/plosone/s/file?id=ba62/PLOSOne_formatting_sample_title_authors_affiliations.pdf>

2. Please note that funding information should not appear in any section or other areas of your manuscript. We will only publish funding information present in the Funding Statement section of the online submission form. Please remove any funding-related text from the manuscript.

3. Thank you for stating the following financial disclosure:

“Funding

This research was supported by: JSPS KAKENHI (JP20K06699) and Scientific Research on Innovative Area (grant No.JP16H01459) to DB, JSPS KAKENHI (JP15K07289) to KY, JSPS KAKENHI (JP21H05659) to HN, JSPS KAKENHI (JP21H04977) and JST CREST (JPMJCR15O1) to HK. Molecular biology equipment was available at Tsukuba-Plant Innovation Research Center (T-PIRC) at the University of Tsukuba.”

Please state what role the funders took in the study.  If the funders had no role, please state: "The funders had no role in study design, data collection and analysis, decision to publish, or preparation of the manuscript."

If this statement is not correct you must amend it as needed.

Please include this amended Role of Funder statement in your cover letter; we will change the online submission form on your behalf.

4. Please expand the acronym “JSPS and JST” (as indicated in your financial disclosure) so that it states the name of your funders in full.

This information should be included in your cover letter; we will change the online submission form on your behalf.

Updated Financial Disclosure and Role of Funders Statement (also included in the cover letter)

Funding

This research was supported by the Japan Society for the Promotion of Science (JSPS) KAKENHI (JP20K06699) and Scientific Research on Innovative Area (grant No. JP16H01459) to DB; JSPS KAKENHI (JP15K07289) to KY; JSPS KAKENHI (JP21H05659) to HN; and JSPS KAKENHI (JP21H04977), the Japan Science and Technology Agency (JST) CREST (JPMJCR15O1) and a Joint Usage program at Centre for Ecological Research, Kyoto University to HK. Molecular biology equipment was available at Tsukuba-Plant Innovation Research Centre (T-PIRC) at the University of Tsukuba. The funders had no role in study design, data collection and analysis, decision to publish, or preparation of the manuscript

5. We note that your Data Availability Statement is currently as follows: All relevant data are within the manuscript and in Supporting Information files.

Please confirm at this time whether or not your submission contains all raw data required to replicate the results of your study. Authors must share the “minimal data set” for their submission. PLOS defines the minimal data set to consist of the data required to replicate all study findings reported in the article, as well as related metadata and methods (https://journals.plos.org/plosone/s/data-availability#loc-minimal-data-set-definition).

For example, authors should submit the following data:

- The values behind the means, standard deviations and other measures reported;

- The values used to build graphs;

- The points extracted from images for analysis.

Authors do not need to submit their entire data set if only a portion of the data was used in the reported study.

If your submission does not contain these data, please either upload them as Supporting Information files or deposit them to a stable, public repository and provide us with the relevant URLs, DOIs, or accession numbers. For a list of recommended repositories, please see <https://journals.plos.org/plosone/s/recommended-repositories>.

To share the “minimal data set” , we have now prepared the file S10 “All supporting data” with all raw data requested, which we uploaded together in the Supporting Information files.

If there are ethical or legal restrictions on sharing a de-identified data set, please explain them in detail (e.g., data contain potentially sensitive information, data are owned by a third-party organization, etc.) and who has imposed them (e.g., an ethics committee). Please also provide contact information for a data access committee, ethics committee, or other institutional body to which data requests may be sent. If data are owned by a third party, please indicate how others may request data access.

This does not apply.

6. We note that Figure 1 and S9 in your submission contain map images which may be copyrighted. All PLOS content is published under the Creative Commons Attribution License (CC BY 4.0), which means that the manuscript, images, and Supporting Information files will be freely available online, and any third party is permitted to access, download, copy, distribute, and use these materials in any way, even commercially, with proper attribution. For these reasons, we cannot publish previously copyrighted maps or satellite images created using proprietary data, such as Google software (Google Maps, Street View, and Earth). For more information, see our copyright guidelines: http://journals.plos.org/plosone/s/licenses-and-copyright.

 We require you to either (1) present written permission from the copyright holder to publish these figures specifically under the CC BY 4.0 license, or (2) remove the figures from your submission:

 1. You may seek permission from the original copyright holder of Figure 1 and S9 to publish the content specifically under the CC BY 4.0 license.

 We recommend that you contact the original copyright holder with the Content Permission Form (http://journals.plos.org/plosone/s/file?id=7c09/content-permission-form.pdf) and the following text:

“I request permission for the open-access journal PLOS ONE to publish XXX under the Creative Commons Attribution License (CCAL) CC BY 4.0 (http://creativecommons.org/licenses/by/4.0/). Please be aware that this license allows unrestricted use and distribution, even commercially, by third parties. Please reply and provide explicit written permission to publish XXX under a CC BY license and complete the attached form.”

Please upload the completed Content Permission Form or other proof of granted permissions as an "Other" file with your submission.

 In the figure caption of the copyrighted figure, please include the following text: “Reprinted from [ref] under a CC BY license, with permission from [name of publisher], original copyright [original copyright year].”

 2. If you are unable to obtain permission from the original copyright holder to publish these figures under the CC BY 4.0 license or if the copyright holder’s requirements are incompatible with the CC BY 4.0 license, please either i) remove the figure or ii) supply a replacement figure that complies with the CC BY 4.0 license. Please check copyright information on all replacement figures and update the figure caption with source information. If applicable, please specify in the figure caption text when a figure is similar but not identical to the original image and is therefore for illustrative purposes only.

The following resources for replacing copyrighted map figures may be helpful:

 USGS National Map Viewer (public domain): http://viewer.nationalmap.gov/viewer/

The Gateway to Astronaut Photography of Earth (public domain): http://eol.jsc.nasa.gov/sseop/clickmap/

Maps at the CIA (public domain): https://www.cia.gov/library/publications/the-world-factbook/index.html and https://www.cia.gov/library/publications/cia-maps-publications/index.html

NASA Earth Observatory (public domain): http://earthobservatory.nasa.gov/

Landsat: http://landsat.visibleearth.nasa.gov/

USGS EROS (Earth Resources Observatory and Science (EROS) Center) (public domain): http://eros.usgs.gov/#

Natural Earth (public domain): <http://www.naturalearthdata.com/>

We removed the images from Fig 1E and S9. The actual physical locations are given in the methods and thus images did not add more information.

7. We note that Figure S1 and S8 in your submission contain copyrighted images. All PLOS content is published under the Creative Commons Attribution License (CC BY 4.0), which means that the manuscript, images, and Supporting Information files will be freely available online, and any third party is permitted to access, download, copy, distribute, and use these materials in any way, even commercially, with proper attribution. For more information, see our copyright guidelines: http://journals.plos.org/plosone/s/licenses-and-copyright.

 We require you to either (1) present written permission from the copyright holder to publish these figures specifically under the CC BY 4.0 license, or (2) remove the figures from your submission:

 1. You may seek permission from the original copyright holder of Figure S1 and S8 to publish the content specifically under the CC BY 4.0 license.

We recommend that you contact the original copyright holder with the Content Permission Form (http://journals.plos.org/plosone/s/file?id=7c09/content-permission-form.pdf) and the following text:

“I request permission for the open-access journal PLOS ONE to publish XXX under the Creative Commons Attribution License (CCAL) CC BY 4.0 (http://creativecommons.org/licenses/by/4.0/). Please be aware that this license allows unrestricted use and distribution, even commercially, by third parties. Please reply and provide explicit written permission to publish XXX under a CC BY license and complete the attached form.”

 Please upload the completed Content Permission Form or other proof of granted permissions as an "Other" file with your submission.

 In the figure caption of the copyrighted figure, please include the following text: “Reprinted from [ref] under a CC BY license, with permission from [name of publisher], original copyright [original copyright year].”

 2. If you are unable to obtain permission from the original copyright holder to publish these figures under the CC BY 4.0 license or if the copyright holder’s requirements are incompatible with the CC BY 4.0 license, please either i) remove the figure or ii) supply a replacement figure that complies with the CC BY 4.0 license. Please check copyright information on all replacement figures and update the figure caption with source information. If applicable, please specify in the figure caption text when a figure is similar but not identical to the original image and is therefore for illustrative purposes only.

We believe that figures S1 and S8 are mistakenly flagged as copyrighted because we produced them.

8. If the reviewer comments include a recommendation to cite specific previously published works, please review and evaluate these publications to determine whether they are relevant and should be cited. There is no requirement to cite these works unless the editor has indicated otherwise.

This does not apply.

Additional Editor Comments:

The paper makes a significant contribution by formalizing the Thermal Memory Interval (TMI) as a transferable metric and extending it to E. japonicum. The research conducted produced field expression datasets which are rare. I agree with the comments raised by both reviewers. The manuscript would benefit from a clearer conceptual framework that explicitly addresses dial up regulation and its role within the proposed model as well description of E. japonicum as species in the context to flowering genes, especially FLC and FT copies.

[Note: HTML markup is below. Please do not edit.]

Please find these points addressed in detail in the reviewers comments below.

Reviewers' comments:

Reviewer's Responses to Questions

**Comments to the Author**

1. Is the manuscript technically sound, and do the data support the conclusions?

The manuscript must describe a technically sound piece of scientific research with data that supports the conclusions. Experiments must have been conducted rigorously, with appropriate controls, replication, and sample sizes. The conclusions must be drawn appropriately based on the data presented.

Reviewer #1: Yes

Reviewer #2: Partly

2. Has the statistical analysis been performed appropriately and rigorously?

Reviewer #1: Yes

Reviewer #2: Yes

3. Have the authors made all data underlying the findings in their manuscript fully available?

The [PLOS Data policy](http://www.plosone.org/static/policies.action#sharing) requires authors to make all data underlying the findings described in their manuscript fully available without restriction, with rare exception (please refer to the Data Availability Statement in the manuscript PDF file). The data should be provided as part of the manuscript or its supporting information, or deposited to a public repository. For example, in addition to summary statistics, the data points behind means, medians and variance measures should be available. If there are restrictions on publicly sharing data—e.g. participant privacy or use of data from a third party—those must be specified.

Reviewer #1: Yes

Reviewer #2: Yes

4. Is the manuscript presented in an intelligible fashion and written in standard English?

PLOS ONE does not copyedit accepted manuscripts, so the language in submitted articles must be clear, correct, and unambiguous. Any typographical or grammatical errors should be corrected at revision, so please note any specific errors here.

Reviewer #1: Yes

Reviewer #2: Yes

5. Review Comments to the Author

Please use the space provided to explain your answers to the questions above. You may also include additional comments for the author, including concerns about dual publication, research ethics, or publication ethics. (Please upload your review as an attachment if it exceeds 20,000 characters)

**Reviewer #1:**

This manuscript presents a comprehensive comparative field study of Arabidopsis halleri subsp. gemmifera and Eutrema japonicum, two perennial Brassicaceae species with contrasting life histories. By integrating two years of high-resolution field gene-expression data with temperature records, the authors analyze seasonal dynamics of the VIN3–FLC–FT regulatory network and introduce the Thermal Memory Interval (TMI) as a quantitative and potentially transferable metric of cellular memory. Overall, this work makes an important contribution to our understanding of long-term epigenetic memory under natural conditions.
However, several issues require clarification and improvement before the manuscript can be considered for publication.

Major

**R1-A. While the manuscript provides extensive analysis and discussion of “dial down” regulation (i.e., suppression of flowering via vernalization), the perspective on “dial up” regulation is insufficiently developed.
If “dial down” corresponds to vernalization-induced repression, how is “dial up” controlled? What molecular or environmental cues trigger the shift from dial down to dial up? How is the endpoint of dial down determined? The manuscript would benefit from a clearer conceptual framework that explicitly addresses dial up regulation and its role within the proposed model.**

**All line numbers cited in our responses below refer to the clean version of the revised manuscript ('Manuscript' file).**

**ANSWER**

We thank the reviewer for this insightful comment. We agree that providing a robust perspective on "dial-up" regulation is essential for the readers of our manuscript. While the field of vernalization research has evolved along parallel—and sometimes unreconciled—axes (annual vs. perennial, lab vs. field, and theoretical vs. experimental), we have previously synthesized these findings into a unified yet transitional model (Buzas et al 2021). To address this in the current manuscript, we have integrated the “Flowering Season-Meter” framework to provide the necessary conceptual home for these observations. This framework explains the transitions between all four seasonal expression quadrants—*FLC* Maximum, Dial-down, *FLC* Minimum, and Dial-up—as a dynamic interaction between a *cis*-acting Polycomb/Trithorax chromatin switch (conserved across life histories) and diffusible *trans*-acting environmental sensors (which provide discrete differences between life histories). From this integrated perspective, dial-up and dial-down are viewed as biologically inseparable components of a continuous cycle (Minimum—Dial-up—Maximum—Dial-down) governed by a single bistable memory system (PcG/trxG-controlled).

We have now modified the Introduction to reflect how this research has evolved historically. We also highlight that the four-phase model emerged both from annual life history studies integrating theoretical-mathematical models with experimental evidence and from field studies in perennial *A. halleri*, which reveaed the annual pattern with 4 phases is spread across the year.

Regarding the specific triggers for phase switching, while the precise molecular mechanisms in perennials remain to be fully resolved and that is not in the scope of our current manuscript (limitations of which we have acknowledged within the manuscript), our study formalizes the Thermal Memory Interval (TMI) as a quantitative metric for memory length. While it was previously well-established in *A. halleri* that temperature throughout the year explains ~80% of *FLC* expression variance, in this study, we extended this findings to *E. japonicum*, demonstrating that this integrative logic is conserved in a perennial species with a different meristem type. Crucially, we now includ a new predictive model where we asked if we could forecast expression in 2021 based on a model trained on 2016–2018 data. The critical transition points—specifically the shift to dial-up—were well-captured using past temperature data alone (Spearman’s ), again strengthening the notion the "trigger" is the plant reaching a specific threshold in its integrated thermal history. Additionally, our work confirms some previous candidate *trans*-acting factors regulating discrete phases of the FLC seasonmeter, as previously noted in the manuscript. One is the seasonal withdrawal of *VIN3* in late spring which serves as a permissive condition for reinstating high *FLC* levels. Second, we noted that species-specific differences in the rate of *FLC* up-regulation correspond to differences in *FT* peak shapes. This is consistent with a known feedback loop where *FT*-mediated pathways may repress *FLC*, thereby modulating the kinetics of the dial-up phase.

**REVISION**

**Conceptual Framework and Historical Perspective (Introduction)**

- **Lines 84–89:**
  - **Text Extract:** "As a powerful paradigm for long-term epigenetic celular memory, vernalization derives its strength from a multi-dimensional research framework, achieved by combining molecular genetic dissection with theoretical modelling, contrasting the distinct evolutionary versions of cellular memory in annual and perennial life histories, and translating laboratory findings within complex natural environments (7). These diverse perspectives are yet to be fully synthesized, and they currently offer distinct observational perspectives on cellular memory."
- **Lines 90–110:**
  - **Text Extract:** " Laboratory assays in the annual life history of Arabidopsis thaliana (A. thaliana) provide a classic model of cellular memory in vernalization, viewed as the persistence of a biological response after the initiating signal disappears. This memory is evident at the organismal level—through the maintenance of vegetative growth after embryo development and the promotion of flowering long after treatment with prolonged low temperatures mimicking winter—and at the transcriptional level, where both the active and repressed states of the central integrator of vernalization, a floral repressor FLOWERING LOCUS C (FLC) are stably maintained across cell divisions. Much like the classic Polycomb and Trithorax Group (PcG/TrxG) gene targets involved in defining Drosophila segment identity (2), FLC exemplifies this principle of persistence: the repressed state remains once the low-temperature signal is removed, while the active state persists even after the initial embryonic activators are no longer present. However, while this laboratory view focuses on evidencing memory through the maintenance of states under simplified, constant temperatures, it may not fully capture the system’s behavior in natural settings where environmental signals fluctuate widely”.
- **Lines 111–131:**
  - **Text Extract:** "Between these poles lie the "dialling" phases—intermediate populations where reversible switching between states enables quantitative shifts in total transcript levels. During dial-down, prolonged cold progressively biases switching toward the OFF state through the sequential action of trans-acting regulators: VERNALIZATION INSENSITIVE 3 (VIN3;(12)) induction during sustained cold enables Polycomb-mediated silencing and the digital accumulation of OFF states, while FLOWERING LOCUS T (FT; (13)) and associated flowering-time pathways contribute to later-stage reinforcement and consolidation of repression. Conversely, during dial-up, switching is biased toward ON states, a process evidenced in two distinct contexts. In the rare Lov-1 ecotype, FLC reactivates during vegetative growth through cell-autonomous OFF-to-ON transitions, revealing the intrinsic bistability of the cis-chromatin (14) . Similarly, genetic and chromatin analyses indicate that the same process in embryos—traditionally described as "resetting" (15,16)—actually reflects staged de novo activation rather than a simple reversal of silencing: early embryonic activation initiates the resolution of OFF states, which are subsequently amplified and stabilized by developmental inputs (17–19). Consequently, the chromatin switch at FLC expands the classical view of memory as simple persistence; instead, it reveals a system that actively tracks state changes across the life cycle, where dial-up and dial-down are inseparable phases of the same switch modulated by temporally distinct trans-acting regulators (7). This 'Season-Meter' framework unifies research that has largely evolved along dispersed observational axes: laboratory studies of 'dial-down' repression (3,12), embryonic studies of 'resetting' (15–18) and ecological studies of perennial cycles (7,20–22)."

**Triggers for Phase Switching and the Predictive Model**

- **Lines 50–52 (Abstract):**
  - **Text Extract:** "Crucially, a regression model forecasted dynamics in an independent year, showing that integrated thermal history explains the timing of seasonal phase switching across the VIN3–FLC–FT network."
- **Lines 169–174 (Introduction):**
  - **Text Extract:** "First, we combined high-resolution gene expression data with environmental temperature records to calculate TMIs and assess their predictive power. By developing a cross-year predictive model to forecast dynamics in an independent year, we tested the robustness of the TMI as a reproducible biological property and evaluated whether the integrated history of past temperatures was sufficient to explain the timing of seasonal gene expression phase switching observed across the network in nature"
- **Lines 573-581 (Figure legend):**
  - **Text Extract: Fig 6. Linear regression analyses and model-based prediction of mRNA dynamics using the simple moving average (SMA) of past temperature.** (A, B) R2 values from linear regression of mRNA levels against SMAs of daily mean temperature with different window lengths, based on the two-year mRNA dynamics of Eutrema japonicum (A) and Arabidopsis halleri (B) shown in Fig 2. (C) Model predictions of mRNA dynamics in E. japonicum from 28 September 2021 to 25 April 2022 (Fig 5), using the regression model in (A) with the best SMA period (highest R2). Spearman’s correlation coefficients (ρ) between observed and predicted values and their P values are shown. In (A-C), lines and shaded areas represent the median and 95% confidence intervals of 1,000 bootstrap samples, respectively. In (A, B), the median and 95% confidence intervals of the best SMA period are shown below the R2 plots.
- **Lines 584–600 (Results):**
  - **Text Extract:** " Our results provide the first estimates of TMIs for VIN3 and FT in two perennial species, as well as extend the time-integrative concept initially identified at FLC in A. halleri. Our data also independently reproduced the estimates of Aikawa et al. (2010) using a different field census from 2012–2014, yielding a nearly identical TMI range of 43–48 days. These results indicate that TMI is consistent across separate field studies spanning six years. We reasoned that if temperature history is the primary and consistent predictor of transcriptional states across the vernalization cascade, a regression model derived from our initial E. japonicum census (2016–2018) should reliably forecast mRNA dynamics in an independent year (2021–2022). Indeed, the regression model developed in Fig 6A accurately predicted the seasonal dynamics observed in the independent year (Fig 5B) with high accuracy (Spearman’s ρ>0.8; Fig 6C). The critical transition points for EjFLC, EjVIN3, and EjFT were well captured using past temperature data alone, with the exception of the full amplitude of the EjFT peak in March. While seasonal timing was accurately predicted, the amplitude of the EjFT peak was not, which is consistent with the burst-like transcriptional behaviour of EjFT observed in the census data (Fig 2C). Overall, these findings demonstrate that the integrated history of past temperatures is sufficient to explain seasonal phase switching at VIN3, FLC, and FT in nature, and once again that TMI represents a consistent metric of integrative thermal memory."

**Molecular and Environmental Cues (VIN3 and FT)**

- **Lines 408–414 (results, previous text):**
  - **Text Extract:** " Moreover, VIN3 reached its seasonal minimum before FLC attained its maximum, raising the possibility that the absence of VIN3 is a permissive condition for re-establishing high FLC levels. Finally, FLC dial-up kinetics differed between species and were mirrored in FT peak shapes: in E. japonicum, the rapid FLC dial-up coincided with a narrower FT peak, whereas in A.halleri the slower FLC dial-up corresponded to a broader FT peak. This could be consistent with a FT-FLC feedback loop evidenced in the winter annual life history (44,45). "

**R1-B. In the transfer experiments from Ikawa to Tsukuba conducted in September and October, the authors need to more clearly articulate their interpretation of why flowering time differs between these treatments.
Why does VIN3 expression differ in December between the September- and October-transferred plants?
Why does VIN3 induction occur so early in plants transferred in September and October?
These observations are intriguing but currently under-discussed. A more explicit explanation or hypothesis is necessary.**

**ANSWER**

We thank the reviewer for these observations and agree that the differences in flowering outcome and the early and divergent *EjVIN3* expression in the transfer experiments require clearer interpretation. We have addressed this by refining our data reporting and clarifying our current understanding of these dynamics.

We have more clearly articulated the flowering outcomes in the Results. While native E. japonicum requires vernalization and flowers in spring, 70% of plants in the September transfer failed to flower (with 30% flowering late), and flowering was completely absent in the October transfer (Fig. 5A). Thus, flowering in both transfer experiments differed substantially from the native conditions. We restricted our interpretation of these phenotypes to experimentally validated gene functions: *EjFLC* act as floral repressor and *EjFT* as a floral promoter. The observed flowering outcomes are consistent with sustained *EjFLC* expression and insufficient *EjFT* induction relative to Ikawa controls (Fig. 5C, D).

We agree that the early induction of *EjVIN3* and the divergence in its expression levels in December are intriguing. We have added a statement clarifying that these patterns may arise when plants are exposed to non-native environmental conditions outside the forest understory of Ikawa, where both temperature and light regimes are typically dampened. Transfer to higher lights and warmer conditions may perturb the integration of thermal and phoperiod cues with the circadian clock, which is —known to regulate *VIN3* (Kyung et al 2022)—thereby permitting earlier induction of VIN3 and contributing to the expression differences observed.We present this as a plausible explanation rather than a definitive mechanism.

Importantly, the primary purpose of the transfer experiments was to perturb the regulatory network using non-native but natural temperature trajectories at different stages of FLC regulation. We were specifically interested in whether long-term trends could be detected and whether a conserved feature between the two perennials—the “domino effect” linking VIN3, FLC, and FT—was maintained across the regulatory cascade, rather than fully resolving the mechanistic basis of each individual regulatory shift.

**REVISION**

**Flowering Outcomes and Interpretation (Methods and Results)**

- **Lines 191–193:**
  - **Text Extract:** **“…**where plants grow under **partial canopy cover in a forest understory environment**, in a flat area with water running down during restricted times of the year.”
- **Lines 518–520:**
  - **Text Extract:** "Flowering differed from native conditions in both transfers: in the September transfer, 70% of plants failed to flower and 30% flowered late, whereas in the October transfer, no plants flowered at all (Fig 5A). ".

**Early Induction and Divergence of *EjVIN3* (Results)**

- **Lines 539–546:**
  - **Text Extract:** "Notably, *EjVIN3* diverged 2–3 months after transfer. In addition, *VIN3* exhibited earlier induction in the transfer experiments than what is typically observed in native populations as well as divergent expression levels between the two transfer groups in December (Fig 5B). This divergence may reflect exposure to non-native environmental conditions, including differences in both temperature and light between the forest understory, canopy-filtered environment at Ikawa and the open-field, full-sun conditions at Tsukuba, as well as may minor variation in sampling time within the defined collection windows, given that VIN3 is regulated by the circadian clock (49)"

R1-C. Because one of the two species (Arabidopsis halleri) has already been reported in similar contexts, it is especially important to provide sufficient basic information for the other species, Eutrema japonicum, in the Introduction.

**ANSWER**

We agree with the reviewer and have expanded the Introduction to provide additional background information on Eutrema japonicum. In addition, we revised the Results section associated with Fig. 1A, B and the Methods section to better contextualize this species, in response to this comment and to related points raised in R1-D,E and R2-A.

REVISION

**Expanded Background (Introduction)**

- **Lines 155–161:**
  - **Text Extract:** "To establish this foundation, we introduced Eutrema japonicum (E. japonicum), a divergent evergreen perennial relative of A. halleri. The E. japonicum genome was recently sequenced (25,26) and revealed an allotetraploid structure where core flowering regulators—including EjFLC, EjVIN3, and EjFT—are present as a single locus per subgenome. This species has a strict requirement for vernalization to initiate flowering (27,28): direct evidence for this requirement is seen in laboratory settings, where specific cold treatments efficiently induce flowering (28), and in agricultural observations where plants remain entirely vegetative throughout the following year if they fail to perceive a sufficient seasonal cooling window (27)"
- **Lines 294–299 (Figure 1B Legend):**
  - **Text Extract:** "(B) General E. japonicum biology. Rosette leaves (pale green) emerge from the central uppermost tiers of the stem. Cauline leaves (yellow) on inflorescence stems emerge from distinct lower tiers and are thus easily distinguished spatially from rosette leaves. Another tier level at the soil interface maintains some meristems in vegetative phase (indeterminate type) to produce new rosette leave (dark green) for clonal propagation. Photographs of flowering A. halleri and E. japonicum can be found in S1 Fig”

**Detailed Genomic Information (Methods)**

- **Lines 191–193:**
  - **Text Extract:** "...where plants grow under partial canopy cover in a forest understory environment, in a flat area with water running down during restricted times of the year."
- **Lines 223–230:**
  - **Text Extract:** "To determine gene copy numbers, BLAST searches were performed using Arabidopsis thaliana sequences from TAIR (AT5G10140.1 for AtFLC, AT5G57380 for AtVIN3, and AT1G65480 for AtFT) as queries against the haplotype-resolved assembly of the E. japonicum ‘Magic’ genome. Results confirmed that EjFLC and EjVIN3 are each present as a single locus per subgenome, located on chromosomes A05 (Accession: CP163465.1) and B05 (Accession: CP163472.1). Similarly, EjFT exists as a single locus per subgenome on chromosomes A06 (Accession: CP163466.1) and B06 (Accession: CP163473.1)."

R1-D.

How many copies of FLC and other key genes exist in the E. japonicum genome?
Are EjFLC1 and EjFLC2 alleles or paralogs (L293)?
EjVIN3 and EjFT are referred to without plural suffixes—should these be considered single-copy genes?
Is this genomic information already known, or is it currently unresolved?

ANSWER

We thank the reviewer for requesting clarification on this point. Following a comprehensive BLAST analysis against the recently released haplotype-resolved, chromosome-level genome assembly of Eutrema japonicum (cultivar ‘Magic’) (Tanaka et al., 2024), we confirmed that EjFLC, EjVIN3, and EjFT are present as single-copy genes per subgenome. As E. japonicum is an allotetraploid, this corresponds to two homeologous copies genome-wide, one in each of the A and B subgenomes. We found no evidence for additional paralogous duplications within either subgenome.

Accordingly, the variants previously referred to as EjFLC1 and EjFLC2 correspond to these subgenome homeologs rather than to distinct paralogous loci. To resolve the inconsistency noted by the reviewer and to align the manuscript with this genomic evidence, we have standardized the terminology throughout the text and figures to use the singular gene designation (e.g., EjFLC). We have updated the Materials and Methods section to explicitly describe this genomic context.

REVISION

**Gene Copy Numbers and Allotetraploid Structure**

- **Lines 156–158 (Introduction):**
  - **Text Extract:** "The E. japonicum genome was recently sequenced (25,26) and revealed an allotetraploid structure where core flowering regulators—including EjFLC, EjVIN3, and EjFT—are present as a single locus per subgenome"
- **Lines 223–230:**
  - **Text Extract:** "To determine gene copy numbers, BLAST searches were performed using Arabidopsis thaliana sequences from TAIR (AT5G10140.1 for AtFLC, AT5G57380 for AtVIN3, and AT1G65480 for AtFT) as queries against the haplotype-resolved assembly of the E. japonicum ‘Magic’ genome. Results confirmed that EjFLC and EjVIN3 are each present as a single locus per subgenome, located on chromosomes A05 (Accession: CP163465.1) and B05 (Accession: CP163472.1). Similarly, EjFT exists as a single locus per subgenome on chromosomes A06 (Accession: CP163466.1) and B06 (Accession: CP163473.1)."

**Clarification of EjFLC1/EjFLC2 as Homeologues**

- **Lines 319–323 (Results):**
  - **Text Extract:** " To characterize the FLC orthologues in E. japonicum in the Ikawa ecotype, we first sequenced cDNA clones spanning the start and stop codons of EjFLC sampled throughout the year. Our sequence analysis identified two types of clones with six single nucleotide polymorphisms resulting in two amino acid differences. The BLAST searches against the “Mazuma” ecotype (25) indicated that they correspond to the A and B subgenomes (S2A Fig). "
- **Lines 300–302 (Figure 1 Legend):**
  - **Text Extract:** "(D) Complementation assay with two Eutrema japonicum FLC genes (EjFLC-A and EjFL-B) in the Arabidopsis thaliana flc3Col-0,..."

R1-E
Furthermore, although results suggest that E. japonicum has a low-temperature requirement, the Introduction does not clearly state whether vernalization is strictly required for flowering induction. Does E. japonicum fail to flower without exposure to low temperature? Are there previous studies addressing this point, or is this still unknown?
These issues are mentioned fragmentarily in the Results, but they should be systematically introduced and clearly explained in the Introduction.

ANSWER

We apologize for this oversight. We extended the paragraph of the introduction to more systematically introduce information about *E. japonicum*. We now clearly explain that *E. japonicum* has strict vernalization requirement based on two studies published in Japanese.

REVISION

**Vernalization Requirement (Introduction)**

- **Lines 158–162:**
  - **Text Extract:** "This species has a strict requirement for vernalization to initiate flowering (27,28): direct evidence for this requirement is seen in laboratory settings, where specific cold treatments efficiently induce flowering (28), and in agricultural observations where plants remain entirely vegetative throughout the following year if they fail to perceive a sufficient seasonal cooling window (27)"

Minor
R1-F.

Does Fig. 1E indicate that flowering time is not delayed in the 40-individual group? If so, what is the proposed explanation for this result? The biological meaning of this observation should be clarified.

ANSWER
We thank the reviewer for requesting this clarification. We have added an explanation for the possible biological meaning.

REVISION

- **Lines 327–331:**
  - **Text Extract:** " While in the majority of independent transgenic lines the T-DNA insertions may have generated insufficiently high expression and the flowering delay was not present or weak (Figure 1D, lines 13 to 39), other lines clearly delayed flowering and, furthermore, 14% of flc3Col-0 35S:EjFLC-A and 18% of flc3Col-0 35S:EjFLC-B did not flower in the first 100 days of the experiment (Fig 1D, lines 40-50). "

R1-G.

There are numerous errors in figure numbering and citation that must be carefully corrected.
L226: Why does the manuscript start with Fig. 6?
Fig. 4A is not cited in the text.
L427: “Fig. 5C” appears to refer to Fig. 3C.
All figure numbers should be checked for consistency between text and figures.
L470–472: It is unclear which month’s FLC expression level is being discussed.
L472: “Fig. 1E” may be an incorrect citation.
In addition to these examples, the manuscript contains many typographical errors and citation mistakes. These issues require careful and thorough revision.

We thank the reviewer for pointing out these issues and sincerely apologize for the errors in figure numbering, citation, and clarity. We have performed a comprehensive revision of the manuscript to ensure that all figures are cited in sequential order and that all figure references accurately correspond to the appropriate data panels.

- The manuscript appeared to begin with Fig. 6 because this figure was first cited in the Materials and Methods section to describe the bootstrap analysis used for estimating full-network TMIs. We have revised the text so that figures are now introduced sequentially within the main body of the Results.
- **Figure 4A:** We have updated the Results text to explicitly cite Fig. 4A, which presents the linear regression analysis of *EjFLC* dialing phases, and ensured that it is properly integrated into the narrative.
- **L470–472:** This section has been rewritten
- **L472:** The citation originally listed as Fig. 1E is correct in substance but was renumbered to Fig. 1D following figure reorganization. The sentence refers to the complementation assay supporting the reported observation, and the citation has been updated accordingly.

In addition, we carefully reviewed the entire manuscript to correct typographical errors, citation inconsistencies, and cross-referencing mistakes throughout the text and figures.

REVISION

Corrected spelling errors are shown by track changes thoughout the manuscript.

- The citation "(Fig 6)" which appeared in the original draft was deleted to prevent the manuscript from "starting" with Figure 6.
- **Lines 355–375 (Results):** Figures are now introduced sequentially starting with **Fig 1A,B** (line **264**)
- **Line 491:** "...14–15 days in E. japonicum and 29–44 days in A.halleri (**Fig 4A, B right**)."
- **Line 493:** "...57–107 days, respectively (**Fig 4A, B left**)."
- **Lines 461–463:** "We note that the timepoints for ChIP assays in Fig 3 correspond with those for gene expression analysis in Fig 5 for September, October, November, February, March, and April (also indicated in Table S1)."
- **EjFLC Nomenclature:** References to the plural "**EjFLCs**" were standardized to the singular "**EjFLC**" to reflect the genomic evidence that it is a single locus per subgenome.
- **Species Prefix:** The original typo "**WjPP2A3**" (original L113) was corrected to "**EjPP2A3**" to match the *Eutrema japonicum* prefix used throughout the paper.
- **Homeologue Naming:** References to **EjFLC1/EjFLC2** were updated to **EjFLC-A/EjFLC-B** to accurately represent the subgenome origin.

Reviewer #2:

The paper makes a significant contribution by formalizing the Thermal Memory Interval (TMI) as a transferable metric and extending it to E. japonicum, VIN3, and FT. Long-term field expression datasets are rare, and most of the claims were promising. However, several methodological concerns, interpretive overreaches, and presentation issues need to be addressed.
Major concerns:

R2-A

Figure 1, would benefit from the inclusion of actual photographs of both species alongside or replacing the current schematic drawings. In particular, E. japonicum is introduced here for the first time as a vernalization model, and readers unfamiliar with this species would benefit from seeing the plant in its natural field setting. Real images would also more effectively communicate the spatial distinction between rosette and cauline leaves that is central to the experimental design. More importantly, the figure as currently presented misses an opportunity to visually highlight what is arguably the manuscript's most significant methodological advance the fact that the same vegetative rosette leaf tissue is used to track FLC expression continuously across all four regulatory phases, including both dial-down in autumn and dial-up in spring, within a single cell lineage.

ANSWER

We thank the reviewer for this constructive suggestion. While we acknowledge the value of actual photographs, we believe that schematic drawings remain the most effective primary tool for communicating relevant aspects of the distinct perennial life histories in this study to a general audience, for the following reasons:

- It is often difficult to distinguish fine anatomical structures in field photographs. Background interference, overlapping vegetation, and physical damage from weather or insects can obscure the key morphological features we are tracking, making them difficult for a reader to isolate.
- Because the various developmental stages—from vegetative growth to full inflorescence—do not occur simultaneously in nature, schematics allow us to present a comprehensive composite of the plant’s annual life cycle that would be impossible to capture in a single field photograph.
- Given PLOS ONE’s broad readership, we believe it is essential to provide an explicit interpretive framework for readers from outside the plant field. A non-expert cannot be expected to instinctively distinguish between overlapping structures—such as the spatial distinction between rosette and cauline leaves—in a raw photograph. Our schematics are designed to “teach” the reader how to navigate the plant's architecture, ensuring they can accurately identify the vegetative cell lineage we are tracking, which is fundamental to understanding our expression and TMI data.

We have re-drawn the schematics (Fig 1A, B) to highlight the spatial distinction between rosette and cauline leaves in colors, emphasizing their differences in size and nonoverlapping position. These modifications underscore our most significant methodological advance: the ability to track *FLC* expression continuously across all four regulatory phases within the same vegetative cell lineage (rosette leaves) in *E.japonicum*.

To answer the request for "real world" evidence, we introduced photographs of a *E. japonicum* in flowering stage in Fig S1D, next to photographs of *A. halleri*.

**REVISION**

- **Lines 285–299:**
  - **Text Extract:** " Fig 1. Perennial life history in two divergent Brassicaceae perennial life histories, E. japonicum vernalization response and transgenic assay in Arabidopsis thaliana (A) General Arabidopsis halleri subsp. gemmifera biology. Rosette leaves (pale green) largely disappear during the peak spring flowering season as vegetative growth converts into reproductive growth. At this time, most of leaves at ground level are basal cauline leaves (yellow). Later, all reproductive meristems revert to vegetative growth (determinate type), giving rise to aerial rosettes (dark green) for clonal propagation. This illustration is a composite image where structures from different developmental stages are drawn together for conceptual clarity; this configuration is not found in natural settings at any one time. (B) General E. japonicum biology. Rosette leaves (pale green) emerge from the central uppermost tiers of the stem. Cauline leaves (yellow) on inflorescence stems emerge from distinct lower tiers and are thus easily distinguished spatially from rosette leaves. Another tier level at the soil interface maintains some meristems in vegetative phase (indeterminate type) to produce new rosette leave (dark green) for clonal propagation. Photographs of flowering A. halleri and E. japonicum can be found in S1 Fig "
- **Lines 839–841:**
  - **Text Extract:** " S1 Fig. …(D). Flowering stage in Eutrema japonicum. Representative plants from April 2022 from the flowering subset of the Ikawa to Tsukuba September transfer experiment (also see Fig 5, S8 Fig). "

R2-B

The paper uses "cellular memory" to describe two mechanistically distinct phenomena: (a) persistence of a chromatin state after the inducing signal disappears (classic epigenetic memory, demonstrated in lab studies of FLC), and (b) integration of fluctuating environmental signals over a time window (what TMI actually measures). These are related but not equivalent. A gene could have a long TMI simply because its regulatory inputs are lagged, without any intrinsic memory mechanism. The Introduction sets this distinction up well (lines 40-44) but the Results and Conclusions repeatedly slide between these usages in ways that obscure what is actually being claimed.

**ANSWER**

We appreciate the reviewer drawing attention to the distinction between the persistence of a chromatin state and the integration of fluctuating signals over time. In our view, the current scientific literature has not yet resolve whether these represent mechanistically distinct forms of memory or are simply different ways of observing and quantifying the same underlying regulatory system.

Even though the research field has not reached a consensus on a unified definition, we agree that clarifying these concepts in the current manuscript is essential for scientific precision. To this end, we have adjusted our formulations and replaced the general term “cellular memory” with the more specific “time-integrative memory” in seven instances where the text refers to the phenomenon captured by the Thermal Memory Interval (TMI). This terminological shift explicitly distinguishes the integrative mode of memory observed in natural, fluctuating environments from the classic view of memory as simple persistence after the disappearance of an inducing signal.

**REVISION**

- **Line 497 (Results):**
  - **Text Extract:** “Thus, TMIs provide a quantitative parameter of **time-integrative memory** length that aligns with known regulatory mechanisms but can be derived from expression and environmental data alone.”
- **Line 603 (Results):**
  - **Text Extract:** “FLC has long been recognized as a striking case of **time-integrative memory**, integrating past temperatures over ~6 weeks (20).”
- **Line 500 (Results):**
  - **Text Extract:** “In this way, TMIs extend mechanistic insights into a generalizable and comparative measure of **time-integrative memory** across contexts.”
- **Line 611 (Results):**
  - **Text Extract:** “While TMIs provide a powerful and generalizable metric for **time-integrative memory**, their limitations should be acknowledged.”
- **Lines 619 (Discussion):**
  - **Text Extract:** “Building on the distributed memory observed in the VIN3–FLC–FT network, TMIs offer a versatile framework to explore how broadly **time-integrative memory** operates across other genes, species, and environmental contexts.”
- **Lines 633(Conclusions):**
  - **Text Extract:**“Importantly, TMIs provide a unified readout of **time-integrative memory** without prior mechanistic knowledge.”
- **Lines ~455–462 (Conclusions):**
  - **Text Extract:** “Because the TMI can be derived directly from high-resolution expression data alongside other dense datasets—such as environmental, anatomical, or physiological measurements—this approach offers a comparative framework for studying **time-integrative memory** across genes, species, and contexts.”

R2-C

Lines 427, 431, 432, and 433 in the Results all refer to "Fig 5C" when the data being discussed clearly belongs to Fig 3C and 3D (the ChIP-qPCR data). This suggests a figure numbering error during revision and needs careful checking throughout the manuscript.

ANSWER

We thank the reviewer for this observation. Actually, in the forementioned Results section we refer to both Fig 3 (the chromatin data) and Fig 5 (the corresponding gene expression data) as the Sep to Nov and Feb to Apr represent the equivalent timepoints. To clarify this,we corrected citations in Results. We also note that the repository of sample, section “Transfer from Ikawa Mountain Center to Tsukuba outdoor” also contains this information.

REVISION

- **Lines 461-463 (Results):**
  - **Text Extract:** “We note that the timepoints for ChIP assays in Fig 3 correspond with those for gene expression analysis in Fig 5 for September, to November and February to April (also indicated in Table S1)”
- **Lines 469 (Results):**
  - **Text Extract:** “…expression (Fig 3C; Ikawa control in Fig 5C)…”
- **Lines 475 (Results):**
  - **Text Extract:** “…transcription-and decreased in spring during dial-up (Fig 3C and D, 5C).”

R2-D
The authors cannot distinguish EjFLC1 from EjFLC2 by qRT-PCR (treated as "EjFLCs" sum) because the sequences are too similar. The ChIP amplicons are designed from 100% identity regions. This means the chromatin profiling in Fig 3 represents an unknown mixture of the two paralogues' chromatin states. If EjFLC1 and EjFLC2 are differentially regulated at the chromatin level, which cannot be excluded, the Fig 3 data could reflect an average of two distinct chromatin dynamics rather than a coherent locus-level signal. This limitation needs to be clearly stated alongside the chromatin data. Perhaps suggest using AS-realtime PCR to identify the allelic expression difference between the two paralogs.
It is quite interesting that the authors did not test the vernalization responsiveness of the E. japonicum FLC prolongs; this would help in understanding the FLC downregulation and upregulation experiments and improve robustness. Currently, it is difficult to determine if the E. japonicum FLC prolongs are vernalization responsive.

ANSWER

We thank the reviewer for these insightful comments. We would like to clarify that the two copies previously referred to in the manuscript are **homeologues,** located on the A and B subgenomes of the allotetraploid *Eutrema japonicum*, rather than paralogues. Given their shared evolutionary origin, similar regulatory behavior between these loci is expected.

To assess this, we analyzed the relative contribution of both homeologues through cDNA sequencing at six time points across the year (Supp Fig S3). This analysis revealed no detectable bias in expression between the two copies at any time point. Based on this consistent pattern, we consider allele-specific qRT-PCR unlikely to change the interpretation of total *EjFLC* expression dynamics, and therefore did not pursue this further.

Regarding vernalization responsiveness, our transfer experiments provide evidence for vernalization responsiveness. Plants transferred to constant 4 °C during winter exhibited strong downregulation of EjFLC within four weeks (Fig. 3A). These observations make it likely that *EjFLC* homeologues are both vernalization-responsive.

We acknowledge, however, that homeologue-specific chromatin dynamics cannot be resolved with our current experimental design. As the ChIP amplicons were designed in regions of 100% sequence identity, the chromatin profiles shown in Fig. 3 represent the combined signal from both homeologues and thus reflect their average epigenetic state. While the similar expression patterns suggest comparable regulation, we cannot exclude homeologue-specific differences at the chromatin level.We have made changes in the manuscript text to acknodegle these limitations, as suggested by the reviewer.

REVISION

- **Lines 437-439 (Results):**
  - **Text Extract:** “Conversely, exposure to 4 °C caused a rapid *EjFLC* decline in winter- consistent with classical vernalization responsiveness- but had no effect in spring until four weeks later.”
- **Lines 482-485(Results):**
  - **Text Extract:** “We note that, as ChIP amplicons were designed in regions of 100% sequence identity, the chromatin profiles in Fig. 3 represent the combined signal from both *EjFLC* homeologues and therefore reflect their average epigenetic state; locus-specific differences cannot be excluded.”

R2-E

The finding that H3K36me3 at the distal nucleation region DNR (ChIP IX) increases during autumn dial-down and decreases during spring dial-up opposite to transcription (lines 432-438) is genuinely interesting and potentially important. This is consistent with antisense COOLAIR transcription, which originates from the 3' end of FLC and is elevated during vernalization. The authors note the pattern but dismiss it in one sentence. Given that antisense transcription from the dNR is mechanistically central to FLC silencing in Arabidopsis thaliana (Hepworth et al., 2018, Chen & Penfield, 2018), this presents an opportunity to considerably strengthen the mechanistic framework. I suggest if it is possible, they should measure the hot air and cool air levels and see how it fits with the diurnal state.

ANSWER

We thank the reviewer for this insightful observation regarding the H3K36me3 patterns at the distal nucleation region (dNR). While we agree that measuring antisense transcripts (COOLAIR) and their diurnal fluctuations could provide a deeper mechanistic layer, we believe these experiments are beyond the scope of the current study for several reasons:

- Study Objective: Our primary goal was to formalize the Thermal Memory Interval (TMI) as a quantifiable metric and to evaluate its conservation across divergent species, rather than to dissect the specific molecular mechanisms of antisense-mediated silencing.
- Technical Limitations: Our existing RNA samples were prepared using standard procedures that were not optimized for the quantification antisense species RNA of less than 200 bp, which were likely excluded during the extraction process.
- Sampling Constraints: Our field census was conducted at single daily timepoints to maintain consistency over the two-year period; consequently, we do not have the diurnal sample sets required to address the reviewer's specific request.
- Complexity in Perennials: The regulation of antisense transcripts in perennial species is currently poorly characterized and represents a highly complex, separate topic that is often controversial even in the annual model *A. thaliana* (Helliwell et al 2011).

We have, however, added a clarifying sentence to the Results section to acknowledge this unresolved mechanistic layer.

REVISION

- **Lines 475-478 (Results):**
  - **Text Extract:** “This pattern may suggest the existence of an antisense-mediated regulatory mechanism (e.g., COOLAIR), which was previously proposed for AhgFLC (21) ,although the specific regulatory mechanisms underlying these dynamics in E. japonicum remain unresolved.”

R2-F
Fig 4 computes TMIs specifically during the dialling phases (dial-up and dial-down separately), while Fig 6 appears to use the full two-year time series. The relationship between these two analyses is not clearly explained. If the full-year analysis in Fig 6 averages across dial-up and dial-down which have opposite TMI signatures the resulting TMI could be biologically uninterpretable. The methods state the intervals used (lines 227-232) but the rationale for using different subsets in different figures is not discussed……

ANSWER

We thank the reviewer for the opportunity to clarify the relationship between the analyses in Figures 4 and 6. The rationale for using different data intervals is based on the distinct biological questions addressed.

In case of Figure 4, the separate calculation of TMIs for the “dial-up” and “dial-down” phases was performed to determine if the TMI framework could extract information that is already biologically established. Specifically, previous research had established that the dialling states are distinct in *A. halleri* (Nishio et al 2020) in regards to sensitivity to “warm” and “cold” temperatures and chromatin states. We confirmed these in *E. japonicum* (Fig 3A, B). By demonstrating that the TMI metric can mathematically recover these known seasonal asymmetries, we validated the TMI as a biologically informative parameter, as stated the initial version of manuscript: “Importantly, the distinction in thermal memory intervals between *FLC* dial-up and *FLC* dial-down recapitulates mechanistic differences previously attributed to *cis*-based chromatin and *trans*-acting factors”.

In case of Figure 6, we applied the TMI to the full annual census to provide a unified, transferable parameter for network-scale comparisons across genes and species.We belive that TMI over full annual census is a robust, quantifiable parameter of time-integrative memory within the vernalization network. This was previously supported in the manuscript by experimental evidence underscoring lon-term memory effects (Fig 5 Transfers). In addition, we have introduced a new line of evidence (Fig 6 C). Using the full-year TMIs have allowed us to develop a regression model that accurately forecasted dynamics in an independent year. This demonstrates that despite the phase-specific nuances identified in Figure 4, the integrated history of past temperatures over the full year is sufficient to explain the timing of seasonal phase switching of all three genes in nature.

Thus, Fig. 4 and Fig. 6 are complementary rather than conflicting. Fig. 4 demonstrates that TMIs can differ between regulatory phases when those phases are analyzed in isolation, while Fig. 6 shows that, when considered across complete seasonal cycles, each gene exhibits a characteristic effective integration timescale consistent with its position in the regulatory network. We agree that this distinction could be clearer, and we have clarified in the Results text.

REVISION

- **Lines 50–52 (Abstract):**
  - **Text Extract:** "Crucially, a regression model forecasted dynamics in an independent year, showing that integrated thermal history explains the timing of seasonal phase switching across the VIN3–FLC–FT network."
- **Lines 169–174 (Introduction):**
  - **Text Extract:** "First, we combined high-resolution gene expression data with environmental temperature records to calculate TMIs and assess their predictive power. By developing a cross-year predictive model to forecast dynamics in an independent year, we tested the robustness of the TMI as a reproducible biological property and evaluated whether the integrated history of past temperatures was sufficient to explain the timing of seasonal gene expression phase switching observed across the network in nature”
- **Lines 556-561 (Results):**
  - **Text Extract:** “First, we combined high-resolution gene expression data with environmental temperature records to calculate TMIs and assess their predictive power. By developing a cross-year predictive model to forecast dynamics in an independent year, we tested the robustness of the TMI as a reproducible biological property and evaluated whether the integrated history of past temperatures was sufficient to explain the timing of seasonal gene expression phase switching observed across the network in nature.”
- **Lines 573–582 (Figure legend):**
  - **Text Extract: “Fig 6. Linear regression analyses and model-based prediction of mRNA dynamics using the simple moving average (SMA) of past temperature.** (A, B) R2 values from linear regression of mRNA levels against SMAs of daily mean temperature with different window lengths, based on the two-year mRNA dynamics of Eutrema japonicum (A) and Arabidopsis halleri (B) shown in Fig 2. (C) Model predictions of mRNA dynamics in E. japonicum from 28 September 2021 to 25 April 2022 (Fig 5), using the regression model in (A) with the best SMA period (highest R2). Spearman’s correlation coefficients (ρ) between observed and predicted values and their P values are shown. In (A-C), lines and shaded areas represent the median and 95% confidence intervals of 1,000 bootstrap samples, respectively. In (A, B), the median and 95% confidence intervals of the best SMA period are shown below the R2 plots.”
- **Lines 584–609 (Results):**
  - **Text Extract:** "Our results provide the first estimates of thermal memory intervals for VIN3 and FT in two perennial species, as well as extend the time-integrative concept initially identified at FLC in A. halleri. Our data also independently reproduced the estimates of Aikawa et al. (2010) using a different field census from 2012–2014, yielding a nearly identical TMI range of 43–48 days. These results indicate that TMI is consistent across separate field studies spanning six years. We reasoned that if temperature history is the primary and consistent predictor of transcriptional states across the vernalization cascade, a regression model derived from our initial wasabi census (2016–2018) should reliably forecast mRNA dynamics in an independent year (2021–2022). Indeed, the regression model developed in Fig. 6C accurately predicted the seasonal dynamics observed in the independent year (Fig. 5B) with high accuracy (Spearman’s ρ; Fig. 6C). The critical transition points for EjFLC, EjVIN3, and EjFT were well captured using past temperature data alone, with the exception of the full amplitude of the EjFT peak in March. While seasonal timing was accurately predicted, the amplitude of the EjFT peak was not, consistent with the burst-like transcriptional behaviour of EjFT observed in the census data (Fig. 2C). Overall, these findings demonstrate that the integrated history of past temperatures is sufficient to explain seasonal phase switching at VIN3, FLC, and FT in nature, and again that TMI represents a consistent metric of integrative thermal memory"

……The claim that EjFT and AhgFT have TMIs of 104-150 days (~21 weeks) is the most striking finding, yet it is the most vulnerable to a trivial alternative explanation. FT is near zero for most of the year and peaks briefly in spring. A long SMA of temperature will correlate well with FT expression simply because it smooths out seasonal variation and tracks the warming trend preceding the FT peak this is a mathematical property of long SMAs applied to seasonal data, not necessarily evidence of cellular memory. The authors need to demonstrate that this long interval cannot be explained by the cascaded delay from VIN3/ FLC/ FT alone, and distinguish between FT intrinsically integrating a 21-week temperature history versus FT inheriting FLC's memory through the regulatory cascade. These are biologically very different claims.

We appreciate the reviewer’s careful consideration of this point and agree that, in principle, long simple moving averages (SMAs) applied to seasonal data can generate spurious correlations with brief expression peaks. Distinguishing biological signal from mathematical artifact is therefore central to our interpretation of long FT thermal memory intervals (TMIs).

Our data argue against the long FT TMIs being a trivial consequence of seasonal smoothing, based on an internal comparative control within the same regulatory network. Both VIN3 and FT exhibit sharply peaked seasonal expression and remain near zero for most of the year (Fig. 2). If long TMIs arose simply from correlating peaked expression with smoothed seasonal temperature trends, then VIN3 would be expected to yield similarly long TMIs. Instead, the TMI framework resolves VIN3 as a rapid temperature sensor, integrating temperature over only 4–6 days in E. japonicum and 1–16 days in A. halleri, while FT integrates over substantially longer intervals of 104–150 days (Fig. 6). This order-of-magnitude divergence despite comparable seasonal expression profiles indicates that long TMIs are not mechanically assigned to genes with brief seasonal peaks.

We also agree that intrinsic versus inherited memory are biologically distinct concepts. We do not claim that FT intrinsically integrates ~21 weeks of temperature at its own locus. Rather, as stated in the manuscript, long TMIs observed at FT likely reflect cumulative thermal information processed upstream in the VIN3–FLC regulatory cascade. In this framework, FT acts as a terminal transcriptional readout of system-level thermal memory rather than an independent molecular memory store. Consistent with this interpretation, we explicitly noted that long TMIs “may reflect cumulative contributions from upstream components rather than intrinsic memory at that gene. TMIs capture memory timescales but do not reveal underlying molecular mechanisms, including *cis*-encoded chromatin states, trans-acting regulation, or feedback loops. Long intervals—such as those observed for FT—may reflect cumulative contributions from upstream components rather than intrinsic memory at that gene.” (Lines 806-812)

R2-G.

To understand the intrinsic speed of Polycomb-mediated silencing, would it be possible to test using the VIN3 inducible construct?

**ANSWER**
We thank the reviewer for these helpful suggestions.

Regarding the possibility of using a VIN3 inducible construct to assay the intrinsic speed of Polycomb-mediated silencing, this approach is not currently feasible in E japonicum. E japonicum is not amenable to stable genetic transformation, and no established protocols exist for generating transgenic lines. Moreover, the field-based design of this study requires plants to complete a full annual cycle under natural conditions, which precludes the use of inducible transgenic systems for both practical and regulatory reasons. For these reasons, our study focuses on endogenous seasonal dynamics of VIN3–FLC regulation rather than experimentally induced perturbations.

Figure quality: All figures are greyscale. My suggestion would be use of colour in Fig 2 (to distinguish species, seasons, or gene expression trajectories) and Fig 5 (where Ikawa vs Tsukuba conditions are plotted together) would substantially improve readability. The current greyscale in Fig 5B-D makes the three experimental series very difficult to distinguish at small print sizes.

We note that Figures 1, 3, and 5 are presented in color, whereas Figures 2, 4, and 6 are rendered in greyscale. In Figure 2 specifically, we revised the layout to improve clarity by simplifying axis elements and standardizing vertical reference lines at three-month intervals across all six panels. This uniform temporal structure facilitates direct comparison of transcriptional trajectories across genes and species. Because each panel in Figure 2 already conveys multiple dimensions of information (gene, species, seasonal phase, and transcriptional dynamics), we found that additional color encoding would reduce, rather than enhance, interpretability.

REVISION

Figure 2 layout was improved.

R2-H

Typos and language errors to address
"Rossette" consistently misspelled in Fig 1 legend (should be "Rosette")

"cellular memory lengh" (line 76) - should be "length"

"hisotires" (line 261) - "histories"

"form high amino acid identity" (line 300) -"from high amino acid identity"

"in of a greater magnitude" (line 321-322) -grammatically incomplete

"the of change of amplitude" (line 491)- grammatically incomplete

"WjPP2A3" (S6 legend, line 756)- should be "EjPP2A3"

ANSWER

We thank the reviewer for carefully identifying these errors. We have corrected all listed typographical and grammatical issues throughout the manuscript and figures, including the misspelling of “Rosette” in Fig. 1, the corrected gene identifier (EjPP2A3 in the S6 legend), and the grammatical errors in the specified lines. We have also carefully proofread the manuscript to ensure that similar errors have been removed.

REVISION

Corrected spelling errors are shown by track changes thoughout the manuscript.

6. PLOS authors have the option to publish the peer review history of their article ([what does this mean?](https://journals.plos.org/plosone/s/editorial-and-peer-review-process#loc-peer-review-history)). If published, this will include your full peer review and any attached files.


If you choose “no”, your identity will remain anonymous but your review may still be made public.


**Do you want your identity to be public for this peer review?** For information about this choice, including consent withdrawal, please see our [Privacy Policy](https://www.plos.org/privacy-policy).

Reviewer #1: No

Reviewer #2: No

[NOTE: If reviewer comments were submitted as an attachment file, they will be attached to this email and accessible via the submission site. Please log into your account, locate the manuscript record, and check for the action link "View Attachments". If this link does not appear, there are no attachment files.]

To ensure your figures meet our technical requirements, please review our figure guidelines: https://journals.plos.org/plosone/s/figures

You may also use PLOS’s free figure tool, NAAS, to help you prepare publication quality figures: https://journals.plos.org/plosone/s/figures#loc-tools-for-figure-preparation.

NAAS will assess whether your figures meet our technical requirements by comparing each figure against our figure specifications.
