# Supplementary material for: Conserved gene- and network-level thermal memory intervals in two divergent perennial crucifers in nature
Source: PLoS One. 2026 Aug 19;21(8):e0336733. doi: 10.1371/journal.pone.0336733 (PMC13489412; doi:10.1371/journal.pone.0336733)
Supplement: Peer review history

# Peer review: Decision Letter 0

**DOI:** 10.1371/journal.pone.0336733.r001

**Authors:** Harsh Raman

17 Mar 2026

Dear Dr. Buzas,

Thank you for submitting your manuscript to PLOS ONE. After careful consideration, we feel that it has merit but does not fully meet PLOS ONE’s publication criteria as it currently stands. Therefore, we invite you to submit a revised version of the manuscript that addresses the points raised during the review process.

The manuscript would benefit from a clearer conceptual framework that explicitly addresses TMI and its role within the proposed model and extending it to E. japonicum. Background information on flowering genes; FLC, FT and VIN will be useful to readers. I agree with both reviewers' comments. Please go through, make corrections and address each point

Please submit your revised manuscript by May 01 2026 11:59PM. If you will need more time than this to complete your revisions, please reply to this message or contact the journal office at plosone@plos.org. When you're ready to submit your revision, log on to https://www.editorialmanager.com/pone/ and select the 'Submissions Needing Revision' folder to locate your manuscript file.

- A letter that responds to each point raised by the academic editor and reviewer(s). You should upload this letter as a separate file labeled 'Response to Reviewers'.
- A marked-up copy of your manuscript that highlights changes made to the original version. You should upload this as a separate file labeled 'Revised Manuscript with Track Changes'.
- An unmarked version of your revised paper without tracked changes. You should upload this as a separate file labeled 'Manuscript'.

If you would like to make changes to your financial disclosure, please include your updated statement in your cover letter. Guidelines for resubmitting your figure files are available below the reviewer comments at the end of this letter.

If applicable, we recommend that you deposit your laboratory protocols in protocols.io to enhance the reproducibility of your results. Protocols.io assigns your protocol its own identifier (DOI) so that it can be cited independently in the future. For instructions see: https://journals.plos.org/plosone/s/submission-guidelines#loc-laboratory-protocols. Additionally, PLOS ONE offers an option for publishing peer-reviewed Lab Protocol articles, which describe protocols hosted on protocols.io. Read more information on sharing protocols at https://plos.org/protocols?utm_medium=editorial-email&utm_source=authorletters&utm_campaign=protocols.

We look forward to receiving your revised manuscript.

Kind regards,

Harsh Raman, Ph.D

Academic Editor

PLOS One

Journal Requirements:

When submitting your revision, we need you to address these additional requirements.

1.Please ensure that your manuscript meets PLOS ONE's style requirements, including those for file naming. The PLOS ONE style templates can be found at

https://journals.plos.org/plosone/s/file?id=wjVg/PLOSOne_formatting_sample_main_body.pdf and

https://journals.plos.org/plosone/s/file?id=ba62/PLOSOne_formatting_sample_title_authors_affiliations.pdf

2. Please note that funding information should not appear in any section or other areas of your manuscript. We will only publish funding information present in the Funding Statement section of the online submission form. Please remove any funding-related text from the manuscript.

3. Thank you for stating the following financial disclosure:

“Funding

This research was supported by: JSPS KAKENHI (JP20K06699) and Scientific Research on Innovative Area (grant No.JP16H01459) to DB, JSPS KAKENHI (JP15K07289) to KY, JSPS KAKENHI (JP21H05659) to HN, JSPS KAKENHI (JP21H04977) and JST CREST (JPMJCR15O1) to HK. Molecular biology equipment was available at Tsukuba-Plant Innovation Research Center (T-PIRC) at the University of Tsukuba.”

Please state what role the funders took in the study.  If the funders had no role, please state: "The funders had no role in study design, data collection and analysis, decision to publish, or preparation of the manuscript."

If this statement is not correct you must amend it as needed.

Please include this amended Role of Funder statement in your cover letter; we will change the online submission form on your behalf.

4. Please expand the acronym “JSPS and JST” (as indicated in your financial disclosure) so that it states the name of your funders in full.

This information should be included in your cover letter; we will change the online submission form on your behalf.

5. We note that your Data Availability Statement is currently as follows: All relevant data are within the manuscript and in Supporting Information files.

Please confirm at this time whether or not your submission contains all raw data required to replicate the results of your study. Authors must share the “minimal data set” for their submission. PLOS defines the minimal data set to consist of the data required to replicate all study findings reported in the article, as well as related metadata and methods (https://journals.plos.org/plosone/s/data-availability#loc-minimal-data-set-definition).

For example, authors should submit the following data:

- The values behind the means, standard deviations and other measures reported;

- The values used to build graphs;

- The points extracted from images for analysis.

Authors do not need to submit their entire data set if only a portion of the data was used in the reported study.

If your submission does not contain these data, please either upload them as Supporting Information files or deposit them to a stable, public repository and provide us with the relevant URLs, DOIs, or accession numbers. For a list of recommended repositories, please see https://journals.plos.org/plosone/s/recommended-repositories.

If there are ethical or legal restrictions on sharing a de-identified data set, please explain them in detail (e.g., data contain potentially sensitive information, data are owned by a third-party organization, etc.) and who has imposed them (e.g., an ethics committee). Please also provide contact information for a data access committee, ethics committee, or other institutional body to which data requests may be sent. If data are owned by a third party, please indicate how others may request data access.

6. We note that Figure 1 and S9 in your submission contain map images which may be copyrighted. All PLOS content is published under the Creative Commons Attribution License (CC BY 4.0), which means that the manuscript, images, and Supporting Information files will be freely available online, and any third party is permitted to access, download, copy, distribute, and use these materials in any way, even commercially, with proper attribution. For these reasons, we cannot publish previously copyrighted maps or satellite images created using proprietary data, such as Google software (Google Maps, Street View, and Earth). For more information, see our copyright guidelines: http://journals.plos.org/plosone/s/licenses-and-copyright.

We require you to either (1) present written permission from the copyright holder to publish these figures specifically under the CC BY 4.0 license, or (2) remove the figures from your submission:

1. You may seek permission from the original copyright holder of Figure 1 and S9 to publish the content specifically under the CC BY 4.0 license.

We recommend that you contact the original copyright holder with the Content Permission Form (http://journals.plos.org/plosone/s/file?id=7c09/content-permission-form.pdf) and the following text:

“I request permission for the open-access journal PLOS ONE to publish XXX under the Creative Commons Attribution License (CCAL) CC BY 4.0 (http://creativecommons.org/licenses/by/4.0/). Please be aware that this license allows unrestricted use and distribution, even commercially, by third parties. Please reply and provide explicit written permission to publish XXX under a CC BY license and complete the attached form.”

Please upload the completed Content Permission Form or other proof of granted permissions as an "Other" file with your submission.

In the figure caption of the copyrighted figure, please include the following text: “Reprinted from [ref] under a CC BY license, with permission from [name of publisher], original copyright [original copyright year].”

2. If you are unable to obtain permission from the original copyright holder to publish these figures under the CC BY 4.0 license or if the copyright holder’s requirements are incompatible with the CC BY 4.0 license, please either i) remove the figure or ii) supply a replacement figure that complies with the CC BY 4.0 license. Please check copyright information on all replacement figures and update the figure caption with source information. If applicable, please specify in the figure caption text when a figure is similar but not identical to the original image and is therefore for illustrative purposes only.

The following resources for replacing copyrighted map figures may be helpful:

USGS National Map Viewer (public domain): http://viewer.nationalmap.gov/viewer/

The Gateway to Astronaut Photography of Earth (public domain): http://eol.jsc.nasa.gov/sseop/clickmap/

Maps at the CIA (public domain): https://www.cia.gov/library/publications/the-world-factbook/index.html and https://www.cia.gov/library/publications/cia-maps-publications/index.html

NASA Earth Observatory (public domain): http://earthobservatory.nasa.gov/

Landsat: http://landsat.visibleearth.nasa.gov/

USGS EROS (Earth Resources Observatory and Science (EROS) Center) (public domain): http://eros.usgs.gov/#

Natural Earth (public domain): http://www.naturalearthdata.com/

7. We note that Figure S1 and S8 in your submission contain copyrighted images. All PLOS content is published under the Creative Commons Attribution License (CC BY 4.0), which means that the manuscript, images, and Supporting Information files will be freely available online, and any third party is permitted to access, download, copy, distribute, and use these materials in any way, even commercially, with proper attribution. For more information, see our copyright guidelines: http://journals.plos.org/plosone/s/licenses-and-copyright.

We require you to either (1) present written permission from the copyright holder to publish these figures specifically under the CC BY 4.0 license, or (2) remove the figures from your submission:

1. You may seek permission from the original copyright holder of Figure S1 and S8 to publish the content specifically under the CC BY 4.0 license.

We recommend that you contact the original copyright holder with the Content Permission Form (http://journals.plos.org/plosone/s/file?id=7c09/content-permission-form.pdf) and the following text:

“I request permission for the open-access journal PLOS ONE to publish XXX under the Creative Commons Attribution License (CCAL) CC BY 4.0 (http://creativecommons.org/licenses/by/4.0/). Please be aware that this license allows unrestricted use and distribution, even commercially, by third parties. Please reply and provide explicit written permission to publish XXX under a CC BY license and complete the attached form.”

Please upload the completed Content Permission Form or other proof of granted permissions as an "Other" file with your submission.

In the figure caption of the copyrighted figure, please include the following text: “Reprinted from [ref] under a CC BY license, with permission from [name of publisher], original copyright [original copyright year].”

2. If you are unable to obtain permission from the original copyright holder to publish these figures under the CC BY 4.0 license or if the copyright holder’s requirements are incompatible with the CC BY 4.0 license, please either i) remove the figure or ii) supply a replacement figure that complies with the CC BY 4.0 license. Please check copyright information on all replacement figures and update the figure caption with source information. If applicable, please specify in the figure caption text when a figure is similar but not identical to the original image and is therefore for illustrative purposes only.

8. If the reviewer comments include a recommendation to cite specific previously published works, please review and evaluate these publications to determine whether they are relevant and should be cited. There is no requirement to cite these works unless the editor has indicated otherwise.

Additional Editor Comments:

The paper makes a significant contribution by formalizing the Thermal Memory Interval (TMI) as a transferable metric and extending it to E. japonicum. The research conducted produced field expression datasets which are rare. I agree with the comments raised by both reviewers. The manuscript would benefit from a clearer conceptual framework that explicitly addresses dial up regulation and its role within the proposed model as well description of E. japonicum as species in the context to flowering genes, especially FLC and FT copies.

[Note: HTML markup is below. Please do not edit.]

Reviewers' comments:

Reviewer's Responses to Questions

**Comments to the Author**

1. Is the manuscript technically sound, and do the data support the conclusions?

Reviewer #1: Yes

Reviewer #2: Partly

**********

2. Has the statistical analysis been performed appropriately and rigorously?

Reviewer #1: Yes

Reviewer #2: Yes

**********

3. Have the authors made all data underlying the findings in their manuscript fully available?

The PLOS Data policy

Reviewer #1: Yes

Reviewer #2: Yes

**********

4. Is the manuscript presented in an intelligible fashion and written in standard English?

Reviewer #1: Yes

Reviewer #2: Yes

**********

Reviewer #1: This manuscript presents a comprehensive comparative field study of Arabidopsis halleri subsp. gemmifera and Eutrema japonicum, two perennial Brassicaceae species with contrasting life histories. By integrating two years of high-resolution field gene-expression data with temperature records, the authors analyze seasonal dynamics of the VIN3–FLC–FT regulatory network and introduce the Thermal Memory Interval (TMI) as a quantitative and potentially transferable metric of cellular memory. Overall, this work makes an important contribution to our understanding of long-term epigenetic memory under natural conditions.

However, several issues require clarification and improvement before the manuscript can be considered for publication.

Major

While the manuscript provides extensive analysis and discussion of “dial down” regulation (i.e., suppression of flowering via vernalization), the perspective on “dial up” regulation is insufficiently developed.

If “dial down” corresponds to vernalization-induced repression, how is “dial up” controlled? What molecular or environmental cues trigger the shift from dial down to dial up? How is the endpoint of dial down determined? The manuscript would benefit from a clearer conceptual framework that explicitly addresses dial up regulation and its role within the proposed model.

In the transfer experiments from Ikawa to Tsukuba conducted in September and October, the authors need to more clearly articulate their interpretation of why flowering time differs between these treatments.

Why does VIN3 expression differ in December between the September- and October-transferred plants?

Why does VIN3 induction occur so early in plants transferred in September and October?

These observations are intriguing but currently under-discussed. A more explicit explanation or hypothesis is necessary.

Because one of the two species (Arabidopsis halleri) has already been reported in similar contexts, it is especially important to provide sufficient basic information for the other species, Eutrema japonicum, in the Introduction.

How many copies of FLC and other key genes exist in the E. japonicum genome?

Are EjFLC1 and EjFLC2 alleles or paralogs (L293)?

EjVIN3 and EjFT are referred to without plural suffixes—should these be considered single-copy genes?

Is this genomic information already known, or is it currently unresolved?

Furthermore, although results suggest that E. japonicum has a low-temperature requirement, the Introduction does not clearly state whether vernalization is strictly required for flowering induction. Does E. japonicum fail to flower without exposure to low temperature? Are there previous studies addressing this point, or is this still unknown?

These issues are mentioned fragmentarily in the Results, but they should be systematically introduced and clearly explained in the Introduction.

Minor

Does Fig. 1E indicate that flowering time is not delayed in the 40-individual group? If so, what is the proposed explanation for this result? The biological meaning of this observation should be clarified.

There are numerous errors in figure numbering and citation that must be carefully corrected.

L226: Why does the manuscript start with Fig. 6?

Fig. 4A is not cited in the text.

L427: “Fig. 5C” appears to refer to Fig. 3C.

All figure numbers should be checked for consistency between text and figures.

L470–472: It is unclear which month’s FLC expression level is being discussed.

L472: “Fig. 1E” may be an incorrect citation.

In addition to these examples, the manuscript contains many typographical errors and citation mistakes. These issues require careful and thorough revision.

Reviewer #2: The paper makes a significant contribution by formalizing the Thermal Memory Interval (TMI) as a transferable metric and extending it to E. japonicum, VIN3, and FT. Long-term field expression datasets are rare, and most of the claims were promising. However, several methodological concerns, interpretive overreaches, and presentation issues need to be addressed.

Major concerns:

Figure 1, would benefit from the inclusion of actual photographs of both species alongside or replacing the current schematic drawings. In particular, E. japonicum is introduced here for the first time as a vernalization model, and readers unfamiliar with this species would benefit from seeing the plant in its natural field setting. Real images would also more effectively communicate the spatial distinction between rosette and cauline leaves that is central to the experimental design. More importantly, the figure as currently presented misses an opportunity to visually highlight what is arguably the manuscript's most significant methodological advance the fact that the same vegetative rosette leaf tissue is used to track FLC expression continuously across all four regulatory phases, including both dial-down in autumn and dial-up in spring, within a single cell lineage.

The paper uses "cellular memory" to describe two mechanistically distinct phenomena: (a) persistence of a chromatin state after the inducing signal disappears (classic epigenetic memory, demonstrated in lab studies of FLC), and (b) integration of fluctuating environmental signals over a time window (what TMI actually measures). These are related but not equivalent. A gene could have a long TMI simply because its regulatory inputs are lagged, without any intrinsic memory mechanism. The Introduction sets this distinction up well (lines 40-44) but the Results and Conclusions repeatedly slide between these usages in ways that obscure what is actually being claimed.

Lines 427, 431, 432, and 433 in the Results all refer to "Fig 5C" when the data being discussed clearly belongs to Fig 3C and 3D (the ChIP-qPCR data). This suggests a figure numbering error during revision and needs careful checking throughout the manuscript.

The authors cannot distinguish EjFLC1 from EjFLC2 by qRT-PCR (treated as "EjFLCs" sum) because the sequences are too similar. The ChIP amplicons are designed from 100% identity regions. This means the chromatin profiling in Fig 3 represents an unknown mixture of the two paralogues' chromatin states. If EjFLC1 and EjFLC2 are differentially regulated at the chromatin level, which cannot be excluded, the Fig 3 data could reflect an average of two distinct chromatin dynamics rather than a coherent locus-level signal. This limitation needs to be clearly stated alongside the chromatin data. Perhaps suggest using AS-realtime PCR to identify the allelic expression difference between the two paralogs.

It is quite interesting that the authors did not test the vernalization responsiveness of the E. japonicum FLC prolongs; this would help in understanding the FLC downregulation and upregulation experiments and improve robustness. Currently, it is difficult to determine if the E. japonicum FLC prolongs are vernalization responsive.

The finding that H3K36me3 at the distal nucleation region DNR (ChIP IX) increases during autumn dial-down and decreases during spring dial-up opposite to transcription (lines 432-438) is genuinely interesting and potentially important. This is consistent with antisense COOLAIR transcription, which originates from the 3' end of FLC and is elevated during vernalization. The authors note the pattern but dismiss it in one sentence. Given that antisense transcription from the dNR is mechanistically central to FLC silencing in Arabidopsis thaliana (Hepworth et al., 2018, Chen & Penfield, 2018), this presents an opportunity to considerably strengthen the mechanistic framework. I suggest if it is possible , they should measure the hot air and cool air levels and see how it fits with the diurnal state.

Fig 4 computes TMIs specifically during the dialling phases (dial-up and dial-down separately), while Fig 6 appears to use the full two-year time series. The relationship between these two analyses is not clearly explained. If the full-year analysis in Fig 6 averages across dial-up and dial-down which have opposite TMI signatures the resulting TMI could be biologically uninterpretable. The methods state the intervals used (lines 227-232) but the rationale for using different subsets in different figures is not discussed.

The claim that EjFT and AhgFT have TMIs of 104-150 days (~21 weeks) is the most striking finding, yet it is the most vulnerable to a trivial alternative explanation. FT is near zero for most of the year and peaks briefly in spring. A long SMA of temperature will correlate well with FT expression simply because it smooths out seasonal variation and tracks the warming trend preceding the FT peak this is a mathematical property of long SMAs applied to seasonal data, not necessarily evidence of cellular memory. The authors need to demonstrate that this long interval cannot be explained by the cascaded delay from VIN3/ FLC/ FT alone, and distinguish between FT intrinsically integrating a 21-week temperature history versus FT inheriting FLC's memory through the regulatory cascade. These are biologically very different claims.

To understand the intrinsic speed of Polycomb-mediated silencing, would it be possible to test using the VIN3 inducible construct?

Figure quality: All figures are greyscale. My suggestion would be use of colour in Fig 2 (to distinguish species, seasons, or gene expression trajectories) and Fig 5 (where Ikawa vs Tsukuba conditions are plotted together) would substantially improve readability. The current greyscale in Fig 5B-D makes the three experimental series very difficult to distinguish at small print sizes.

Typos and language errors to address

"Rossette" consistently misspelled in Fig 1 legend (should be "Rosette")

"cellular memory lengh" (line 76) - should be "length"

"hisotires" (line 261) - "histories"

"form high amino acid identity" (line 300) -"from high amino acid identity"

"in of a greater magnitude" (line 321-322) -grammatically incomplete

"the of change of amplitude" (line 491)- grammatically incomplete

"WjPP2A3" (S6 legend, line 756)- should be "EjPP2A3"

**********

what does this mean?). If published, this will include your full peer review and any attached files.

If you choose “no”, your identity will remain anonymous but your review may still be made public.

**Do you want your identity to be public for this peer review?**  For information about this choice, including consent withdrawal, please see our Privacy Policy

Reviewer #1: No

Reviewer #2: No

**********

[NOTE: If reviewer comments were submitted as an attachment file, they will be attached to this email and accessible via the submission site. Please log into your account, locate the manuscript record, and check for the action link "View Attachments". If this link does not appear, there are no attachment files.]

To ensure your figures meet our technical requirements, please review our figure guidelines: https://journals.plos.org/plosone/s/figures

You may also use PLOS’s free figure tool, NAAS, to help you prepare publication quality figures: https://journals.plos.org/plosone/s/figures#loc-tools-for-figure-preparation.

NAAS will assess whether your figures meet our technical requirements by comparing each figure against our figure specifications.

# Author comment: Author response to Decision Letter 1

**DOI:** 10.1371/journal.pone.0336733.r002

26 May 2026

The following is an extract of the "response to reviewers™ file where all responses are marked in red.

5. Review Comments to the Author

Please use the space provided to explain your answers to the questions above. You may also include additional comments for the author, including concerns about dual publication, research ethics, or publication ethics. (Please upload your review as an attachment if it exceeds 20,000 characters)

Reviewer #1:

This manuscript presents a comprehensive comparative field study of Arabidopsis halleri subsp. gemmifera and Eutrema japonicum, two perennial Brassicaceae species with contrasting life histories. By integrating two years of high-resolution field gene-expression data with temperature records, the authors analyze seasonal dynamics of the VIN3–FLC–FT regulatory network and introduce the Thermal Memory Interval (TMI) as a quantitative and potentially transferable metric of cellular memory. Overall, this work makes an important contribution to our understanding of long-term epigenetic memory under natural conditions.

However, several issues require clarification and improvement before the manuscript can be considered for publication.

Major

R1-A. While the manuscript provides extensive analysis and discussion of “dial down” regulation (i.e., suppression of flowering via vernalization), the perspective on “dial up” regulation is insufficiently developed.

If “dial down” corresponds to vernalization-induced repression, how is “dial up” controlled? What molecular or environmental cues trigger the shift from dial down to dial up? How is the endpoint of dial down determined? The manuscript would benefit from a clearer conceptual framework that explicitly addresses dial up regulation and its role within the proposed model.

All line numbers cited in our responses below refer to the clean version of the revised manuscript ('Manuscript' file).

ANSWER

We thank the reviewer for this insightful comment. We agree that providing a robust perspective on "dial-up" regulation is essential for the readers of our manuscript. While the field of vernalization research has evolved along parallel—and sometimes unreconciled—axes (annual vs. perennial, lab vs. field, and theoretical vs. experimental), we have previously synthesized these findings into a unified yet transitional model (Buzas et al 2021). To address this in the current manuscript, we have integrated the “Flowering Season-Meter” framework to provide the necessary conceptual home for these observations. This framework explains the transitions between all four seasonal expression quadrants—FLC Maximum, Dial-down, FLC Minimum, and Dial-up—as a dynamic interaction between a cis-acting Polycomb/Trithorax chromatin switch (conserved across life histories) and diffusible trans-acting environmental sensors (which provide discrete differences between life histories). From this integrated perspective, dial-up and dial-down are viewed as biologically inseparable components of a continuous cycle (Minimum—Dial-up—Maximum—Dial-down) governed by a single bistable memory system (PcG/trxG-controlled).

We have now modified the Introduction to reflect how this research has evolved historically. We also highlight that the four-phase model emerged both from annual life history studies integrating theoretical-mathematical models with experimental evidence and from field studies in perennial A. halleri, which reveaed the annual pattern with 4 phases is spread across the year.

Regarding the specific triggers for phase switching, while the precise molecular mechanisms in perennials remain to be fully resolved and that is not in the scope of our current manuscript (limitations of which we have acknowledged within the manuscript), our study formalizes the Thermal Memory Interval (TMI) as a quantitative metric for memory length. While it was previously well-established in A. halleri that temperature throughout the year explains ~80% of FLC expression variance, in this study, we extended this findings to E. japonicum, demonstrating that this integrative logic is conserved in a perennial species with a different meristem type. Crucially, we now includ a new predictive model where we asked if we could forecast expression in 2021 based on a model trained on 2016–2018 data. The critical transition points—specifically the shift to dial-up—were well-captured using past temperature data alone (Spearman’s ), again strengthening the notion the "trigger" is the plant reaching a specific threshold in its integrated thermal history. Additionally, our work confirms some previous candidate trans-acting factors regulating discrete phases of the FLC seasonmeter, as previously noted in the manuscript. One is the seasonal withdrawal of VIN3 in late spring which serves as a permissive condition for reinstating high FLC levels. Second, we noted that species-specific differences in the rate of FLC up-regulation correspond to differences in FT peak shapes. This is consistent with a known feedback loop where FT-mediated pathways may repress FLC, thereby modulating the kinetics of the dial-up phase.

REVISION

Conceptual Framework and Historical Perspective (Introduction)

• Lines 84–89:

o Text Extract: "As a powerful paradigm for long-term epigenetic celular memory, vernalization derives its strength from a multi-dimensional research framework, achieved by combining molecular genetic dissection with theoretical modelling, contrasting the distinct evolutionary versions of cellular memory in annual and perennial life histories, and translating laboratory findings within complex natural environments (7). These diverse perspectives are yet to be fully synthesized, and they currently offer distinct observational perspectives on cellular memory."

• Lines 90–110:

o Text Extract: " Laboratory assays in the annual life history of Arabidopsis thaliana (A. thaliana) provide a classic model of cellular memory in vernalization, viewed as the persistence of a biological response after the initiating signal disappears. This memory is evident at the organismal level—through the maintenance of vegetative growth after embryo development and the promotion of flowering long after treatment with prolonged low temperatures mimicking winter—and at the transcriptional level, where both the active and repressed states of the central integrator of vernalization, a floral repressor FLOWERING LOCUS C (FLC) are stably maintained across cell divisions. Much like the classic Polycomb and Trithorax Group (PcG/TrxG) gene targets involved in defining Drosophila segment identity (2), FLC exemplifies this principle of persistence: the repressed state remains once the low-temperature signal is removed, while the active state persists even after the initial embryonic activators are no longer present. However, while this laboratory view focuses on evidencing memory through the maintenance of states under simplified, constant temperatures, it may not fully capture the system’s behavior in natural settings where environmental signals fluctuate widely”.

• Lines 111–131:

o Text Extract: "Between these poles lie the "dialling" phases—intermediate populations where reversible switching between states enables quantitative shifts in total transcript levels. During dial-down, prolonged cold progressively biases switching toward the OFF state through the sequential action of trans-acting regulators: VERNALIZATION INSENSITIVE 3 (VIN3;(12)) induction during sustained cold enables Polycomb-mediated silencing and the digital accumulation of OFF states, while FLOWERING LOCUS T (FT; (13)) and associated flowering-time pathways contribute to later-stage reinforcement and consolidation of repression. Conversely, during dial-up, switching is biased toward ON states, a process evidenced in two distinct contexts. In the rare Lov-1 ecotype, FLC reactivates during vegetative growth through cell-autonomous OFF-to-ON transitions, revealing the intrinsic bistability of the cis-chromatin (14) . Similarly, genetic and chromatin analyses indicate that the same process in embryos—traditionally described as "resetting" (15,16)—actually reflects staged de novo activation rather than a simple reversal of silencing: early embryonic activation initiates the resolution of OFF states, which are subsequently amplified and stabilized by developmental inputs (17–19). Consequently, the chromatin switch at FLC expands the classical view of memory as simple persistence; instead, it reveals a system that actively tracks state changes across the life cycle, where dial-up and dial-down are inseparable phases of the same switch modulated by temporally distinct trans-acting regulators (7). This 'Season-Meter' framework unifies research that has largely evolved along dispersed observational axes: laboratory studies of 'dial-down' repression (3,12), embryonic studies of 'resetting' (15–18) and ecological studies of perennial cycles (7,20–22)."

Triggers for Phase Switching and the Predictive Model

• Lines 50–52 (Abstract):

o Text Extract: "Crucially, a regression model forecasted dynamics in an independent year, showing that integrated thermal history explains the timing of seasonal phase switching across the VIN3–FLC–FT network."

• Lines 169–174 (Introduction):

o Text Extract: "First, we combined high-resolution gene expression data with environmental temperature records to calculate TMIs and assess their predictive power. By developing a cross-year predictive model to forecast dynamics in an independent year, we tested the robustness of the TMI as a reproducible biological property and evaluated whether the integrated history of past temperatures was sufficient to explain the timing of seasonal gene expression phase switching observed across the network in nature"

• Lines 573-581 (Figure legend):

o Text Extract: Fig 6. Linear regression analyses and model-based prediction of mRNA dynamics using the simple moving average (SMA) of past temperature. (A, B) R2 values from linear regression of mRNA levels against SMAs of daily mean temperature with different window lengths, based on the two-year mRNA dynamics of Eutrema japonicum (A) and Arabidopsis halleri (B) shown in Fig 2. (C) Model predictions of mRNA dynamics in E. japonicum from 28 September 2021 to 25 April 2022 (Fig 5), using the regression model in (A) with the best SMA period (highest R2). Spearman’s correlation coefficients (ρ) between observed and predicted values and their P values are shown. In (A-C), lines and shaded areas represent the median and 95% confidence intervals of 1,000 bootstrap samples, respectively. In (A, B), the median and 95% confidence intervals of the best SMA period are shown below the R2 plots.

• Lines 584–600 (Results):

o Text Extract: " Our results provide the first estimates of TMIs for VIN3 and FT in two perennial species, as well as extend the time-integrative concept initially identified at FLC in A. halleri. Our data also independently reproduced the estimates of Aikawa et al. (2010) using a different field census from 2012–2014, yielding a nearly identical TMI range of 43–48 days. These results indicate that TMI is consistent across separate field studies spanning six years. We reasoned that if temperature history is the primary and consistent predictor of transcriptional states across the vernalization cascade, a regression model derived from our initial E. japonicum census (2016–2018) should reliably forecast mRNA dynamics in an independent year (2021–2022). Indeed, the regression model developed in Fig 6A accurately predicted the seasonal dynamics observed in the independent year (Fig 5B) with high accuracy (Spearman’s ρ>0.8; Fig 6C). The critical transition points for EjFLC, EjVIN3, and EjFT were well captured using past temperature data alone, with the exception of the full amplitude of the EjFT peak in March. While seasonal timing was accurately predicted, the amplitude of the EjFT peak was not, which is consistent with the burst-like transcriptional behaviour of EjFT observed in the census data (Fig 2C). Overall, these findings demonstrate that the integrated history of past temperatures is sufficient to explain seasonal phase switching at VIN3, FLC, and FT in nature, and once again that TMI represents a consistent metric of integrative thermal memory."

Molecular and Environmental Cues (VIN3 and FT)

• Lines 408–414 (results, previous text):

o Text Extract: " Moreover, VIN3 reached its seasonal minimum before FLC attained its maximum, raising the possibility that the absence of VIN3 is a permissive condition for re-establishing high FLC levels. Finally, FLC dial-up kinetics differed between species and were mirrored in FT peak shapes: in E. japonicum, the rapid FLC dial-up coincided with a narrower FT peak, whereas in A.halleri the slower FLC dial-up corresponded to a broader FT peak. This could be consistent with a FT-FLC feedback loop evidenced in the winter annual life history (44,45). "

R1-B. In the transfer experiments from Ikawa to Tsukuba conducted in September and October, the authors need to more clearly articulate their interpretation of why flowering time differs between these treatments.

Why does VIN3 expression differ in December between the September- and October-transferred plants?

Why does VIN3 induction occur so early in plants transferred in September and October?

These observations are intriguing but currently under-discussed. A more explicit explanation or hypothesis is necessary.

ANSWER

We thank the reviewer for these observations and agree that the differences in flowering outcome and the early and divergent EjVIN3 expression in the transfer experiments require clearer interpretation. We have addressed this by refining our data reporting and clarifying our current understanding of these dynamics.

We have more clearly articulated the flowering outcomes in the Results. While native E. japonicum requires vernalization and flowers in spring, 70% of plants in the September transfer failed to flower (with 30% flowering late), and flowering was completely absent in the October transfer (Fig. 5A). Thus, flowering in both transfer experiments differed substantially from the native conditions. We restricted our interpretation of these phenotypes to experimentally validated gene functions: EjFLC act as floral repressor and EjFT as a floral promoter. The observed flowering outcomes are consistent with sustained EjFLC expression and insufficient EjFT induction relative to Ikawa controls (Fig. 5C, D).

We agree that the early induction of EjVIN3 and the divergence in its expression levels in December are intriguing. We have added a statement clarifying that these patterns may arise when plants are exposed to non-native environmental conditions outside the forest understory of Ikawa, where both temperature and light regimes are typically dampened. Transfer to higher lights and warmer conditions may perturb the integration of thermal and phoperiod cues with the circadian clock, which is —known to regulate VIN3 (Kyung et al 2022)—thereby permitting earlier induction of VIN3 and contributing to the expression differences observed.We present this as a plausible explanation rather than a definitive mechanism.

Importantly, the primary purpose of the transfer experiments was to perturb the regulatory network using non-native but natural temperature trajectories at different stages of FLC regulation. We were specifically interested in whether long-term trends could be detected and whether a conserved feature between the two perennials—the “domino effect” linking VIN3, FLC, and FT—was maintained across the regulatory cascade, rather than fully resolving the mechanistic basis of each individual regulatory shift.

REVISION

Flowering Outcomes and Interpretation (Methods and Results)

• Lines 191–193:

o Text Extract: “…where plants grow under partial canopy cover in a forest understory environment, in a flat area with water running down during restricted times of the year.”

• Lines 518–520:

o Text Extract: "Flowering differed from native conditions in both transfers: in the September transfer, 70% of plants failed to flower and 30% flowered late, whereas in the October transfer, no plants flowered at all (Fig 5A). ".

Early Induction and Divergence of EjVIN3 (Results)

• Lines 539–546:

o

Attachment Submitted filename: Response to reviewers .docx

# Peer review: Decision Letter 1

**DOI:** 10.1371/journal.pone.0336733.r003

**Authors:** Harsh Raman

13 Jul 2026

Conserved gene- and network-level thermal memory intervals in two divergent perennial crucifers in nature

PONE-D-25-58585R1

Dear Dr. Buzas,

We’re pleased to inform you that your manuscript has been judged scientifically suitable for publication and will be formally accepted for publication once it meets all outstanding technical requirements.

Within one week, you’ll receive an e-mail detailing the required amendments. When these have been addressed, you’ll receive a formal acceptance letter and your manuscript will be scheduled for publication.

An invoice will be generated when your article is formally accepted. Please note, if your institution has a publishing partnership with PLOS and your article meets the relevant criteria, all or part of your publication costs will be covered. Please make sure your user information is up-to-date by logging into Editorial Manager at Editorial Manager® and clicking the ‘Update My Information' link at the top of the page. For questions related to billing, please contact billing support.

If your institution or institutions have a press office, please notify them about your upcoming paper to help maximize its impact. If they’ll be preparing press materials, please inform our press team as soon as possible -- no later than 48 hours after receiving the formal acceptance. Your manuscript will remain under strict press embargo until 2 pm Eastern Time on the date of publication. For more information, please contact onepress@plos.org.

Kind regards,

Harsh Raman, Ph.D

Academic Editor

PLOS One

Additional Editor Comments (optional):

Sorry, it took a long-time.

Reviewers' comments:

Reviewer's Responses to Questions

**Comments to the Author**

Reviewer #1: All comments have been addressed

Reviewer #2: All comments have been addressed

**********

2. Is the manuscript technically sound, and do the data support the conclusions?

Reviewer #1: Yes

Reviewer #2: Yes

**********

3. Has the statistical analysis been performed appropriately and rigorously?

Reviewer #1: Yes

Reviewer #2: Yes

**********

4. Have the authors made all data underlying the findings in their manuscript fully available?

The PLOS Data policy

Reviewer #1: Yes

Reviewer #2: Yes

**********

5. Is the manuscript presented in an intelligible fashion and written in standard English?

Reviewer #1: Yes

Reviewer #2: Yes

**********

Reviewer #1: The revised manuscript has been substantially improved and addresses all of my previous concerns. I have no further comments and recommend its acceptance for publication.

Reviewer #2: I am happy to see that the authors have addressed the comments and provided a brief overview of the technical difficulties encountered in certain experiments.

**********

what does this mean?). If published, this will include your full peer review and any attached files.

If you choose “no”, your identity will remain anonymous but your review may still be made public.

**Do you want your identity to be public for this peer review?**  For information about this choice, including consent withdrawal, please see our Privacy Policy

Reviewer #1: No

Reviewer #2: No

**********

# Editor report: Acceptance letter

**DOI:** 10.1371/journal.pone.0336733.r004

**Authors:** Harsh Raman

PONE-D-25-58585R1

PLOS One

Dear Dr. Buzas,

I'm pleased to inform you that your manuscript has been deemed suitable for publication in PLOS One. Congratulations! Your manuscript is now being handed over to our production team.

At this stage, our production department will prepare your paper for publication. This includes ensuring the following:

* All references, tables, and figures are properly cited

* All relevant supporting information is included in the manuscript submission,

* There are no issues that prevent the paper from being properly typeset

You will receive further instructions from the production team, including instructions on how to review your proof when it is ready. Please keep in mind that we are working through a large volume of accepted articles, so please give us a few days to review your paper and let you know the next and final steps.

Lastly, if your institution or institutions have a press office, please let them know about your upcoming paper now to help maximize its impact. If they'll be preparing press materials, please inform our press team within the next 48 hours. Your manuscript will remain under strict press embargo until 2 pm Eastern Time on the date of publication. For more information, please contact onepress@plos.org.

You will receive an invoice from PLOS for your publication fee after your manuscript has reached the completed accept phase. If you receive an email requesting payment before acceptance or for any other service, this may be a phishing scheme. Learn how to identify phishing emails and protect your accounts at https://explore.plos.org/phishing.

If we can help with anything else, please email us at customercare@plos.org.

Thank you for submitting your work to PLOS ONE and supporting open access.

Kind regards,

PLOS ONE Editorial Office Staff

on behalf of

Dr. Harsh Raman

Academic Editor

PLOS One
